# Structural insights of ZIP4 extracellular domain critical for optimal zinc transport

**DOI:** 10.1038/ncomms11979

**Published:** 2016-06-20

**Authors:** Tuo Zhang, Dexin Sui, Jian Hu

**Affiliations:** 1 Department of Biochemistry and Molecular Biology, Michigan State University, East Lansing, Michigan 48824, USA; 2 Department of Chemistry, Michigan State University, East Lansing, Michigan 48824, USA

## Abstract

The ZIP zinc transporter family is responsible for zinc uptake from the extracellular milieu or intracellular vesicles. The LIV-1 subfamily, containing nine out of the 14 human ZIP proteins, is featured with a large extracellular domain (ECD). The critical role of the ECD is manifested by disease-causing mutations on ZIP4, a representative LIV-1 protein. Here we report the first crystal structure of a mammalian ZIP4-ECD, which reveals two structurally independent subdomains and an unprecedented dimer centred at the signature PAL motif. Structure-guided mutagenesis, cell-based zinc uptake assays and mapping of the disease-causing mutations indicate that the two subdomains play pivotal but distinct roles and that the bridging region connecting them is particularly important for ZIP4 function. These findings lead to working hypotheses on how ZIP4-ECD exerts critical functions in zinc transport. The conserved dimeric architecture in ZIP4-ECD is also demonstrated to be a common structural feature among the LIV-1 proteins.

## Abstract (web summary)

Mutations in the extracellular domain of zinc transporter ZIP4 result in a lethal disorder. Here, the authors report the first crystal structure of ZIP4 extracellular domain, unveiling its unprecedented dimerization and two structural independent subdomains that have crucial roles in zinc transport.

## Graphical abstract

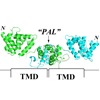

Zinc ion is essential in numerous biological processes for the organisms in all the three kingdoms of life. In humans, zinc is the second most abundant trace element (after iron). Besides the well-established catalytic and structural roles in biomacromolecules, zinc ion has been shown to act as a signalling molecule regulating diverse cellular functions^1^. Accordingly, the intracellular zinc concentration needs to be precisely regulated. In mammals, zinc homeostasis is primarily maintained by two zinc transporter families, ZnT and ZIP. The ZnT family (Solute Carrier 30, SLC30A) facilitates zinc efflux from the cytoplasm^2^,^3^,^4^,^5^; whereas the ZIP family (Zrt/Irt-like Protein, SLC39A) mediates zinc influx from either the extracellular milieu or intracellular vesicles, increasing the cytoplasmic zinc concentration^4^,^5^,^6^,^7^.

A total of 14 human ZIP proteins have been identified based on the sequence similarity to the zinc-regulated transporter 1 and 2 from yeast^6^ and iron-regulated transporter from *Arabidopsis thaliana*^7^, which can be further divided into four subfamilies. The fourth subfamily, which contains nine ZIP proteins, is also called the LIV-1 subfamily^8^. The LIV-1 proteins play pivotal and diverse roles in both physiological and pathological processes. ZIP4 is the exclusive zinc transporter specifically expressed on the apical surface of intestinal epithelium and responsible for dietary zinc absorption^9^. ZIP4 knockout in mouse is embryonic lethal, and loss-of-function mutations of ZIP4 lead to a lethal genetic disorder, Acrodermatitis Enteropathica (AE)^9^,^10^,^11^,^12^. In addition, ZIP4 expression was up-regulated in different types of cancers cells^13^,^14^,^15^, and has been proposed to be a potential drug target against pancreatic cancer^16^,^17^,^18^. The brain specifically expressed ZIP12, the closest homologue of ZIP4, is essential for neuronal system development^19^. ZIP13 mediates intracellular zinc traffic and the mutations lead to spondylocheirodysplastic form of Ehlers–Danlos syndrome, a genetic disorder characterized with defects in connective tissues, bones and teeth^20^,^21^,^22^. ZIP8 is a key player in inflammation^23^ and a recent report established its pathological roles in occurrence and development of osteoarthritis^24^. ZIP6, also called LIV-1, is broadly involved in a variety of cancers, particularly breast cancer^25^,^26^,^27^,^28^.

Structurally, the LIV-1proteins share a similar topology: a transmembrane domain (TMD) with predicted eight transmembrane helices and a large and variable N-terminal extracellular domain (ECD). A highly conserved proline-alanine-leucine (PAL) motif in the ECDs suggests common features in structure and function of the ECDs among the LIV-1 proteins. Remarkably, out of the 15 unambiguous missense AE-causing mutations on ZIP4, seven of them are in the ECD (refs 29, 30), implying that ZIP4-ECD is a functionally important domain. The previous biochemical and cell biological study have shown that disease-causing mutations on ZIP4-ECD affected zinc transport activity, transporter processing and trafficking^31^,^32^. However, the lack of the structural information of ZIP4-ECD greatly hampers the understanding of the underlying mechanism about how ZIP4-ECD exerts critical roles in ZIP4 function.

In this work, we present the first crystal structure of a mammalian ZIP4-ECD at a resolution of 2.8 Å, which reveals an unprecedented dimer centred at the signature PAL motif. Structure-guided mutagenesis and cell-based zinc transport assay, as well as mapping of the disease-causing mutations, indicate that the two structurally independent subdomains in ZIP4-ECD play distinct roles in ZIP4 function. Based on these findings, we propose hypotheses about how ZIP4-ECD acts as a critical accessory domain essential for optimal zinc transport. Structure-based sequence analysis also suggests a common dimeric architecture of the ECDs within the LIV-1 subfamily, shedding light on the structural and mechanistic study of the other LIV-1 proteins.

## Results

### ZIP4-ECD is required for high efficient zinc transport

**Figure 1:**
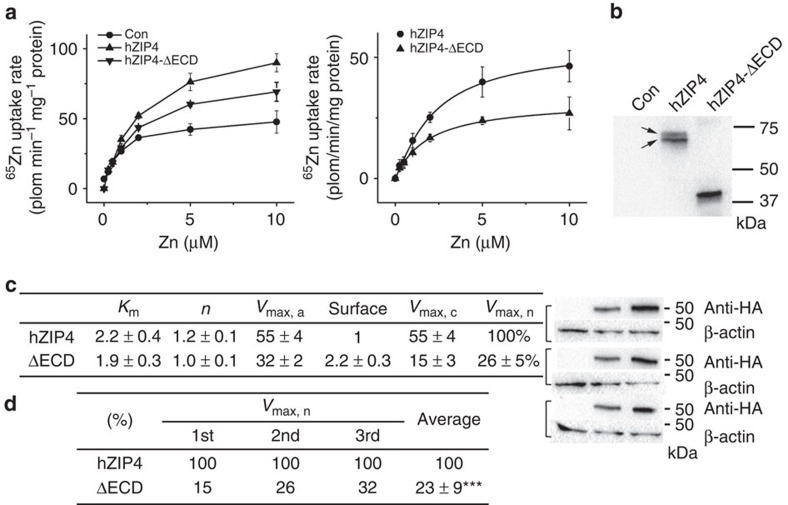
The role of ZIP4-ECD in ZIP4-mediated zinc transport. (**a**) Cell-based zinc uptake assay. HEK293T cells transiently expressing hZIP4 and hZIP4-ΔECD were incubated with the indicated concentrations of ZnCl_2_ (containing 20% ^65^ZnCl_2_) in zinc uptake buffer at 37 °C for 20 min. After rapid washing on ice, the cells were lysed and the amount of zinc was measured using a Gamma counter. The cells transfected with the empty vector were used as control. The raw experimental data (left) and the processed data (right) after subtracting the zinc uptake in cells transfected with empty vector represent the results of one out of three independent experiments. The data from the other two experiments are shown in Supplementary Fig. 1 in Supplementary Information. Each data point represents the mean of three replicates. The error bars indicate 1±s.d. The results of curve fitting using the Hill model are shown in the Table 1 (**c**). (**b**) top: western blot of hZIP4 and hZIP4-ΔECD expressed in HEK293T cells using anti-HA antibody. The glycosylated (upper band) and un-glycosylated (lower band) proteins are indicated by arrows. bottom: comparison of the surface expression levels of hZIP4 and hZIP4-ΔECD with three replicates. The surface expression level is indicated by the surface bound anti-HA antibody. β-actin was detected using anti-β-actin antibody in western blot as loading control. (**c**) Zinc transport kinetic parameters of hZIP4 and hZIP4-ΔECD. The units of *K*_m_ and *V*_max_ are μM and pmol min^−1^ mg^−1^, respectively. To obtain the calibrated *V*_max_ (*V*_max, c_), the apparent *V*_max_ (V_max, a_) calculated from curve fitting (OriginPro 8.5) was divided by the averaged surface expression level determined by Image Lab (Bio Rad) programme. The surface expression level of the corresponding protein is expressed as a ratio relative to that of the wild-type hZIP4. The calibrated *V*_max_s were then normalized by dividing the calibrated *V*_max_ of the wild-type hZIP4 and expressed as percentages. (**d**) Statistical analysis of the normalized *V*_max_s (*V*_max, n_) from three independent experiments. The Student's *t*-test is used to assess significance statistically.***P<0.001.

To investigate the role of ZIP4-ECD, we deleted the entire ECD from human ZIP4 (hZIP4-ΔECD, Δ38–317, the signal peptide was kept for proper localization and orientation in plasma membrane) and compared its zinc transport activity with the full-length ZIP4 (hZIP4) in a cell-based zinc uptake assay. As shown in Fig. 1, cells expressing hZIP4-ΔECD showed much less ^65^zinc uptake than cells expressing the full-length protein. The largely reduced zinc uptake is not due to a significantly reduced surface expression level of hZIP4-ΔECD. In contrast, higher surface expression level for the truncated ZIP4 was observed although the same amounts of plasmid DNA were used in transfection (Fig. 1 and Supplementary Fig. 1), indicating ZIP4-ECD is not required for folding and trafficking of ZIP4-TMD. Curve fitting using the Hill model shows a ∼50% decrease in apparent *V*_max_, whereas no significant changes in *K*_m_ and the Hill coefficient (*n*). After calibration using the quantitated surface expression level of the corresponding construct, our data reveal that ZIP4-ECD deletion leads to a 70–80% decrease of *V*_max_, indicating that ZIP4-ECD is essential for optimal zinc transport. Therefore, ZIP4-ECD promotes the function of the zinc transport machinery, whereas has little contribution to the affinity towards zinc ions.

### Crystal structure determination of a mammalian ZIP4-ECD

To gain the structural insights into the function of ZIP4-ECD, we screened ZIP4-ECDs from different species. Because of the eight invariant cysteine residues in ZIP4-ECD, proteins overexpressed in regular *Escherichia coli* strains were severely aggregated. To facilitate disulfide bond formation and protein folding, the *E. coli* strain Origami was used to produce the proteins. A ZIP4-ECD from *Pteropus Alecto* (black fruit bat, pZIP4-ECD) was found to have ideal behaviour in solution. pZIP4-ECD shares 68% identical residues with human ZIP4-ECD (Supplementary Fig. 2a), and cell-based zinc uptake assay confirmed that the full-length pZIP4 is a functional zinc transporter (Supplementary Fig. 2b).

After extensive screening, pZIP4-ECD was crystallized and the crystal structure was solved using single-wavelength anomalous dispersion data from a crystal of selenomethionine-substituted protein at the resolution of 2.85 Å, and the phase was applied to a native data set with slightly better data quality at 2.8 Å (Table 1 for crystallographic statistics, Supplementary Fig. 3 for stereo view of the 2F_o_−F_c_ electron density map and Supplementary Fig. 4 for anomalous difference Fourier map of selenium atoms).

### Overall structure of pZIP4-ECD

**Figure 2:**
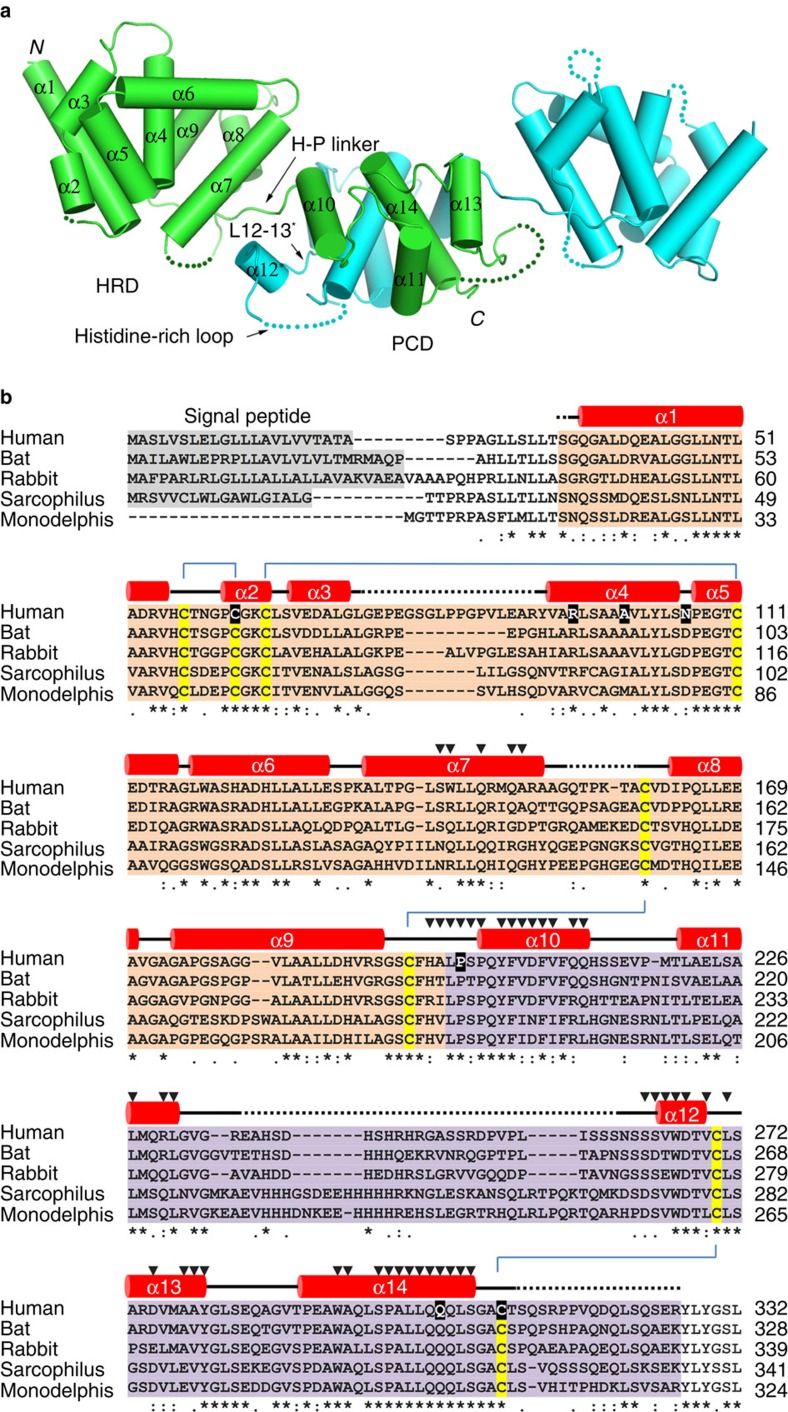
Crystal structure of pZIP4-ECD. (**a**) The structure of pZIP4-ECD dimer. The helices are presented as cylinders. Only the helices in the green protomer are labelled, except that α12 in the blue protomer is labelled. It is because α12 in the green molecule is seriously disordered. The disordered sequences are shown as dashed lines. Key structural elements, including the H-P linker, L12–13 and the histidine-rich loop, are indicted by arrows. (**b**) Multiple sequence alignment of mammalian ZIP4-ECDs. The Genebank codes and NCBI reference sequence codes are: AAH62625 for human, ELK11751 for bat, XP_004580947 for rabbit, XP_003760599 for Sarcophilus and XP_001371869 for Monodelphis. The predicted signal peptides are shown in grey, the HRD in orange and the PCD in purple. The invariant cysteine residues are highlighted in yellow and the disulfide bonds are indicated with blue lines. The residues subjected to mutations in AE are highlighted in black blocks. The residues involved in dimerization are labelled with black triangles. The secondary structures derived from pZIP4-ECD structure are shown above the sequences. The dashed lines indicate the disordered regions.

In one asymmetric unit, two pZIP4-ECD molecules form a homodimer (Fig. 2a). The structure of pZIP4-ECD shows that each protomer has two structurally independent subdomains. The N-terminal subdomain consists of 156 amino acid residues (residues 36–191, orange in Fig. 2b). This subdomain is primarily composed of a cluster of α-helices (α1–9) centred at α4. This subdomain has a quite high α-helical content (73%), and therefore we name this subdomain as helix rich domain (HRD). The C-terminal subdomain (residues 192–322, purple in Fig. 2b) harbours the signature PAL motif and it is named as PAL motif containing domain (PCD). The PCD consists of a pair of helix-turn-helix folds (α10, α11 and α13, α14) and the PAL motif is in the middle of the long α14. A short helix α12 on the side of the PCD is linked to α13 through an extended loop (L12–13). In the PCD, a segment (residues 232–255) between α11 and α12, which is called histidine-rich loop because of a cluster of histidine residues, is severely disordered. Besides a short loop connecting the HRD and the PCD between α9 and α10 (H-P linker), α12* (*means it is from the other protomer) and L12–13* are also involved in communication of the two subdomains. Therefore, we refer to the region consisting of the H–P loop as α12* and L12–13* as the bridging region (Fig. 2a).

The structure of pZIP4-ECD is stabilized by four disulfide bonds (C59–C64, C67–C103, C153–C188 and C266–C305) (Fig. 2b and Supplementary Fig. 5). Because the AE-causing mutations C62R and C309Y (C59 and C305 in pZIP4) eliminate the first and the fourth disulfide bonds, respectively, and the corresponding mutations in mouse ZIP4 resulted in completely diminished ZIP4 glycosylation^32^, these disulfide bonds must be critical for ZIP4 folding.

### Dimerization of pZIP4-ECD

**Figure 3:**
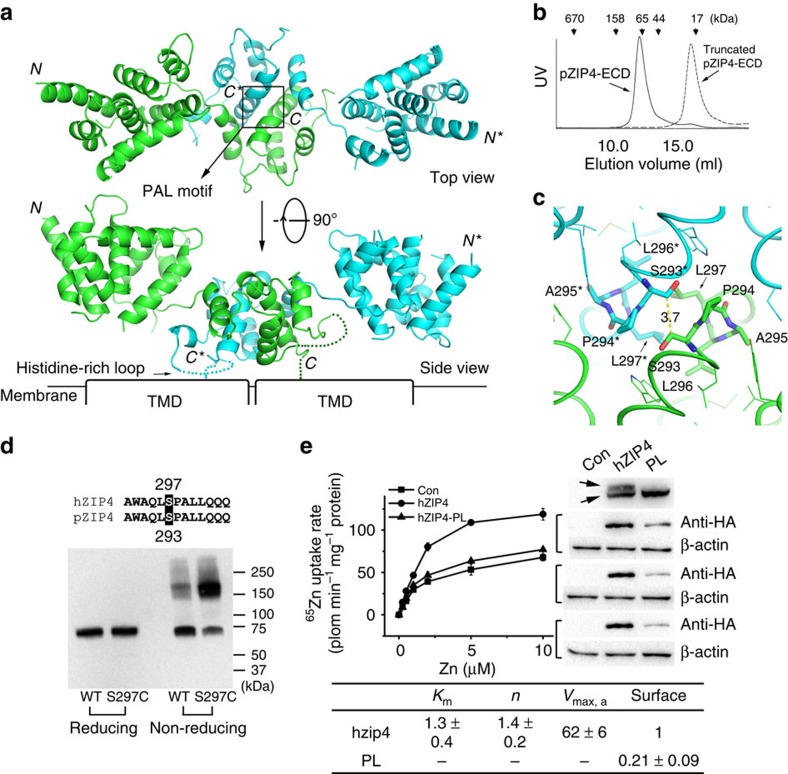
Dimerization of pZIP4-ECD. (**a**) Top view and side view of pZIP4-ECD. The PAL motif at the centre of the dimerization interface is indicated in the top view. The putative membrane surface and the TMD are illustrated in the side view. The disordered histidine rich loop and the linker connecting the ECD and the TMD are depicted as dashed lines. (**b**) Size exclusion chromatography profiles of pZIP4-ECD and a truncated construct terminated at the end of α11. The peaks of the standard proteins eluted on the same column are indicated by arrows labelled with the molecular weights. (**c**) The zoomed-in structure of the PAL motif in the top view. The labelled residues in ^293^SPALL^297^ sequence are shown as stick. The other conserved residues around the PAL motif are shown in line model. (**d**) Western blots of the wild-type hZIP4 and the S297C mutant in the presence (left) and absence (right) of 5% β-mercaptoethanol. As shown in the sequence alignment, S297 in hZIP4 is equivalent to S293 in pZIP4. A 4–20% gradient SDS–PAGE gel was used for better resolution at high molecular weight region. (**e**) Functional characterization of hZIP4 P298A and L300A double mutant (PL). left: zinc uptake assay. The shown data are the results of one out of three independent experiments. The data from the other two experiments are shown in Supplementary Fig. 8a,b. Each data point represents the mean of three replicates. The error bars indicate 1±s.d. right: western blot of the wild-type hZIP4 (WT) and the P298A and L300A double mutant. The glycosylated (upper band) and un-glycosylated (lower band) proteins are indicated by arrows. The surface expression level is indicated by the surface bound anti-HA antibody with three replicates. β-actin was detected using anti-β-actin antibody in western blot as loading control. bottom: zinc transport kinetics using the Hill model. The units of *K*_m_ and *V*_max_ are μM and pmol min^−1^ mg^−1^, respectively. Note that the kinetic parameters of the PL mutant were not measured because the activity is too low.

As shown in Figs 2a and 3a, two pZIP4-ECD molecules form an elongated and wing-shaped dimer: the two PCDs pack together and form a central dimeric module, which is flanked by two HRDs forming the ends of the wing. At the first glance, this unique architecture appears to be a result of domain swapping. Indeed, rotating 180° of the PCD relative to the twofold symmetric axis at the centre of the PCD leads to a perfect overlapping of the two PCDs. However, a close inspection reveals that the H–P linker connecting the HRD and the PCD is too short to re-connect the two subdomains after 180° rotation of the PCD, excluding the possibility of domain swapping. The dimer is as long as 110 Å, whereas the size of the transverse section of the wing is quite small (35 Å × 35 Å). Because the predicted TM1 is 16 residues downstream of the last ordered residue (C305) in pZIP4-ECD structure, the orientation of the dimer relative to the membrane can be deduced as shown in Fig. 3a: the two PCDs are tethered to the TMD with a twofold symmetric axis perpendicular to membrane plane; whereas the two HRDs are hanging over the membrane surface. Notably, the two protomers in one asymmetric unit are not in the same conformation. Structure superimposition of the PCDs reveals that the HRD of molecule B (HRD_B_) rotates, from the position of the HRD_A_, ∼20° relative to the PCD dimer (Supplementary Fig. 6), suggestive of structural flexibility of the bridging region.

All the helices in the PCD are involved in dimerization. Particularly, α14, which is surrounded by the other helices in the PCD (α10–13, α10* and α11*), directly interacts with α14* from the other protomer, playing a central role in dimerization. In the HRD, only α7 is contributive to dimerization through a weak association with α12* in the PCD. For each protomer (287 residues), a total of 44 residues are involved in dimerization (Fig. 2b), which buries an area of 1,534 Å^2^ as measured by PDBePIS^33^. Both polar and non-polar interactions are contributive to dimerization. The estimated free energy (ΔG∼−30.3 kcal mol^−1^) suggests a very stable dimer. Indeed, the elution volume of the purified pZIP4-ECD on size exclusion chromatography indicates an apparent molecular weight of ∼65–70 kDa, suggestive of a dimer in solution (the monomeric protein is 32 kDa) (Fig. 3b, solid line). Consistent with this dimerization model, a construct truncated at the end of α11 (residues 36–233) is eluted as a monomeric protein in size exclusion chromatography (Fig. 3b, dashed line), confirming the essential role of the PCD in dimerization.

### The PAL motif at the dimerization interface

A close inspection at the pZIP4-ECD dimer reveals that the residues at the dimerization interface (particularly those in the PCD) are considerably conserved across species (Fig. 2b). Most interestingly, the absolutely conserved proline residue (P294) in the PAL motif, together with a highly conserved serine residue (S293), are located at the cross of the two α14s which are at the centre of the PCD dimer (Fig. 3c). As a known helix breaker, P294 is responsible for bending of α14, which enlarges the interface of α14 with α10, α11 and α13, and therefore stabilizes the PCD structure. In addition, the near-planar side chains of the P294 and P294* are parallel to each other and facing towards the small side chain of S293* and S293, respectively, facilitating the approaching of the two α14s and stabilizing the dimer. A295 in the PAL motif is on the opposite side of S293 and P294, and surrounded by several highly conserved hydrophobic residues, participating in a hydrophobic core on the dimerization interface. L296 in the PAL motif and the highly conserved L297 are one turn downstream of S293 and P294, and directly contact with L296* and L297* on the dimerization interface. Collectively, the PAL motif at the centre of the dimerization interface seems to play a structural role.

To validate that the observed PCD dimer is biologically relevant, we introduced a cysteine residue at S297 of the full-length hZIP4, which is equivalent to S293 in pZIP4 according to sequence alignment (Fig. 2b), and transiently expressed the mutant protein in HEK293T cells. Because S293 and S293* face to each other and close in space (the distance between the two C_β_s is 3.7 Å), we predicted that the two Cys residues would form an intermolecular disulfide bond. As shown in Fig. 3d, both wild-type ZIP4 and S293C are monomeric in reducing SDS–polyacrylamide gel electrophoresis (SDS–PAGE) with an apparent molecular weight of 70–75 kDa. In contrast, in non-reducing SDS–PAGE, most of the mutant protein form oligomer with an apparent molecular weight ∼150–180 kDa, indicative of a homodimer, whereas the wild-type protein is still primarily monomeric. Zinc uptake assay showed that the S297C mutant ZIP4 exhibits similar zinc transport activity as the wild-type ZIP4 with no defect in surface expression and glycosylation (Supplementary Fig. 7). Therefore, the dramatically increased dimerization of the S297C mutant ZIP4 is not due to mutation induced misfolding. These results indicate that: (1) there is no intermolecular disulfide bond for the wild-type hZIP4, albeit the full-length hZIP4 contains 15 cysteine residues. The small amount of dimer (and even higher-order oligomers shown as smeared bands at high molecular weight region) observed in the non-reducing gel may be due to misfolded protein on membrane protein overexpression; (2) C297 and C297* in the mutant protein are close enough for disulfide bond formation, consistent with the identified dimerization interface in the crystal structure of pZIP4-ECD. Some mutant proteins are still monomeric, probably because that the side chains of the cysteine residues may have alternative orientations (that is, rotamers) unfavourable for disulfide bond formation. Collectively, these data confirm the homodimerization of ZIP4-ECD observed in the crystal structure and unambiguously indicate that the functionally expressed full-length hZIP4 forms a homodimer in cells, but not via any intermolecular disulfide bond.

To examine the importance of the PAL motif for ZIP4 function, we introduced a P298A and L300A double mutation in the PAL motif of hZIP4 and tested zinc transport activity in the cell-based zinc uptake assay. Remarkably, zinc uptake by the cells expressing the double mutant protein is barely higher than the cells transfected with the empty vector (Fig. 3e and Supplementary Fig. 8a,b), which is primarily due to the dramatically reduced surface expression level of the double mutant protein. We noticed that the total expression level of the mutant protein is still comparable with that of the wild-type protein, and therefore, the completely abrogated glycosylation and largely reduced surface expression indicate severe defects in folding and trafficking of the mutant transporter, consistent with a critical structural role of the PAL motif.

### Crucial roles of the HRD and the bridging region

**Figure 4:**
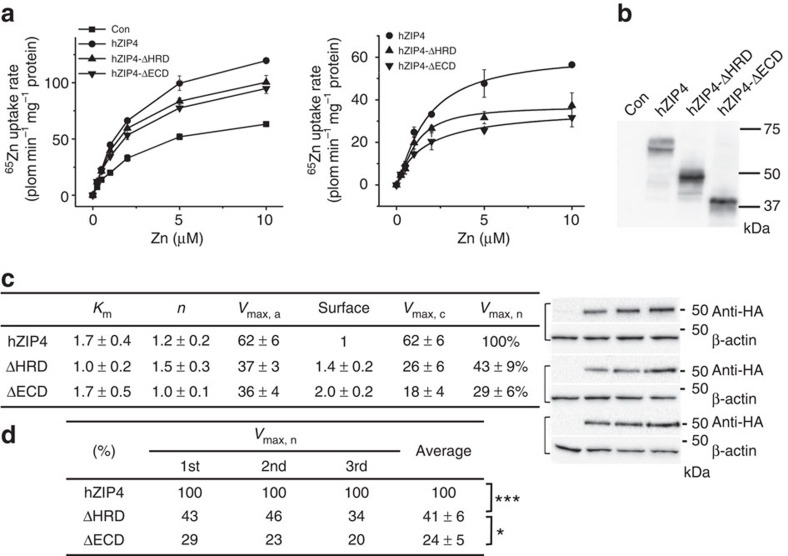
The role of the HRD in zinc transport. (**a**) Zinc uptake assay and curve fitting of hZIP4, hZIP4-ΔHRD and hZIP4-ΔECD expressed in HEK293T cells. left: the raw experimental data; right: the processed data. Each data point represents the mean of three replicates. The error bars indicate 1±s.d. The results of curve fitting using the Hill model are shown in the Table 1 (**c**). (**b**) Western blot of hZIP4, hZIP4-ΔHRD and hZIP4-ΔECD expressed in HEK293T cells and surface expression levels are indicated by the surface bound anti-HA antibody with three replicates. β-actin was detected using anti-β-actin antibody in western blot as loading control. (**c**) Zinc transport kinetic parameters. The shown data are results of one out of three independent experiments. The data from the other two experiments are shown in Supplementary Fig. 8c,d. The units of *K*_m_ and *V*_max_ are μM and pmol min^−1^ mg^−1^, respectively. (**d**) Statistical analysis of the normalized *V*_max_s (*V*_max, n_) from three independent experiments. The Student's *t*-test was used to assess significance statistically. ***P<0.001 and *P<0.05.

A three dimensional protein structure similarity search on the Dali server^34^ shows that the HRD is a novel protein fold. To examine the functionality of the HRD, we made an HRD-deletion construct (hZIP4-ΔHRD, Δ38–96) and compared its activity with hZIP4 and hZIP4-ΔECD. As shown in Fig. 4 and Supplementary Fig. 8c,d, hZIP4-ΔHRD can be well expressed on cell surface, but the calibrated *V*_max_ of hZIP4-ΔHRD is significantly lower than that of hZIP4, and a little bit higher than that of hZIP4-ΔECD, suggesting that both the HRD and the PCD are contributive to optimal zinc uptake.

**Figure 5:**
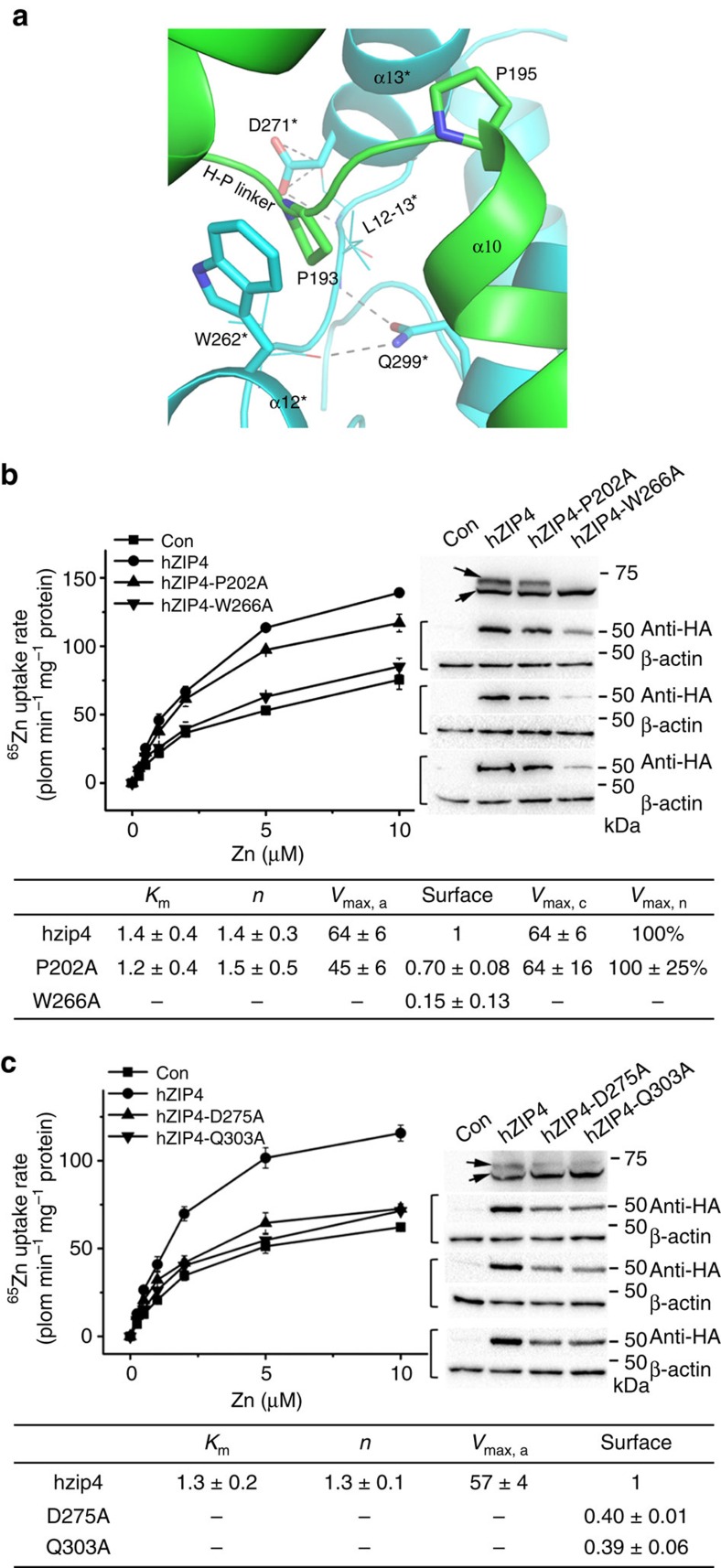
The roles of the bridging region in ZIP4 biogenesis and zinc transport. (**a**) The zoomed-in structure of the bridging region. The key residues are shown in stick mode and other involved residues are shown in line mode. The dashed lines indicate potential hydrogen bonds. In hZIP4, P202 (P195 in pZIP4), W266 (W262 in pZIP4), D275 (D271 in pZIP4) and Q303 (Q299 in pZIP4) are mutated into alanine. (**b**) Functional characterization of P202A and W266A. (**c**) Functional characterization of D275A and Q303A. In both **b** and **c**, the raw experimental data of zinc transport assay (left), and western blots of the mutants in HEK293T cells, surface expression detection and western blots of β-actin (right) are from one representative experiment and the data sets from the other two independent experiments are shown in Supplementary Fig. 8e–h. Zinc transport kinetic parameters and statistical analysis are shown in the tables at the bottom. The units of *K*_m_ and *V*_max_ are μM and pmol min^−1^ mg^−1^, respectively. For W262A, D275A and Q303A, their zinc transport activities are too low to be accurately determined using the current approach. See more detailed data processing in Experimental Procedure and the figure legend of Fig. 1.

As the HRD communicates with the PCD exclusively through the bridging region, we are wondering whether the bridging region plays any role in ZIP4 function. Therefore, we mutated four highly conserved residues (W266, P202, D275 and Q303) into alanine in hZIP4 to disrupt the structure of the bridging region (Fig. 5a). W266* (W262 in pZIP4) on α12* specifically interacts with P200 (P193 in pZIP4) at the H-P linker through side-chain interaction (note that P200L is an AE-causing mutation). P202 (P195 in pZIP4) is the last residue of the H-P linker, immediately preceding α10. D275 (D271 in pZIP4) on α13 associates with L12–13* through its interactions with the conserved S272* (S268 in pZIP4) on L12–13, whereas Q303 (Q299 in pZIP4) is involved in the interaction of α14 with L12–13. As shown in Fig. 5b,c and Supplementary Fig. 8e–h, except for P202A which shows no defects in zinc uptake, glycosylation and surface expression, the other three mutants exhibit very low zinc uptake activity. W266A led to a completely diminished glycosylation and very little surface expression, indicating severe defects in protein folding and trafficking. D275A and Q303A also showed reduced glycosylation level and surface expression, but to a less extent compared with W266A. The very low zinc transport activities of D275A and Q303A suggest their *V*_max_s could be substantially smaller than that of the wild-type protein. Taken together, the bridging region appears to be critical for both ZIP4 folding, trafficking and zinc transport.

### Mapping of the AE-causing mutations

**Figure 6:**
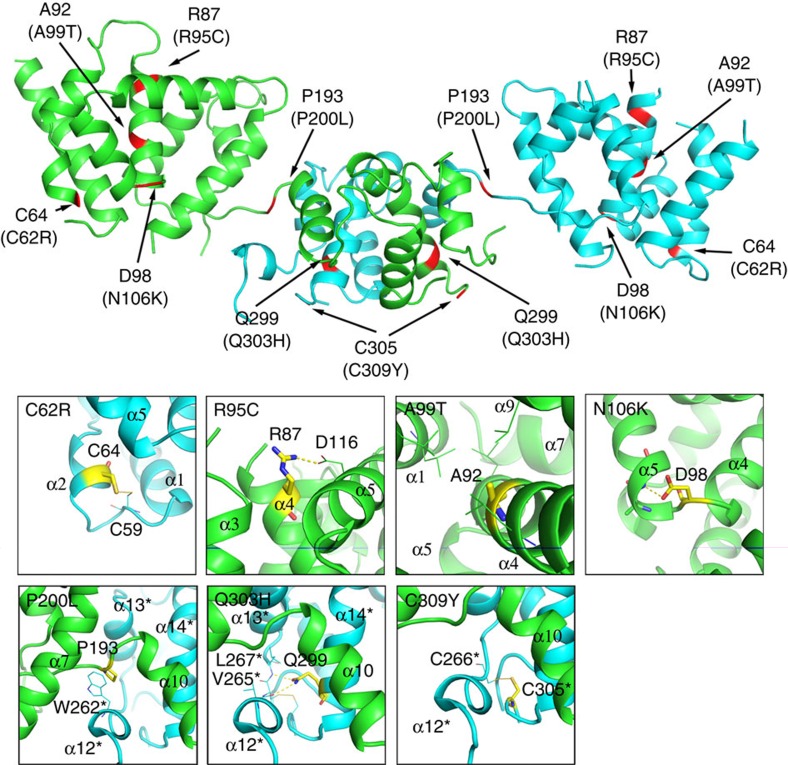
Mapping AE-causing mutations on the structure of pZIP4-ECD dimer. The residues subjected to AE-causing mutations are highlighted in red. Mutations in human ZIP4 are shown in the brackets and the corresponding residues in pZIP4-ECD are indicated above the brackets. The zoomed-in structures in the proximity of these residues are shown in the small figures.

The high homology between pZIP4-ECD and hZIP4-ECD (68% identical residues, Supplementary Fig. 2a) enables us to map the AE-causing mutations on the structure of pZIP4-ECD (Fig. 6 and Fig. 2b), providing an opportunity to probe the structure–function relationship.

In the HRD, besides C62R (C59 in pZIP4) affecting ZIP4 folding^32^, the other three AE-causing mutations [R95C (R87 in pZIP4), A99T (A92 in pZIP4) and N106K (D98 in pZIP4)] are either on or close to α4 at the centre of the HRD: the highly conserved R87 forms a salt bridge with another conserved residue D116 on α6, stabilizing the packing of α6 on α4; A92 on α4 is buried in a highly hydrophobic environment; whereas the conserved D98 appears to be important to maintain the sharp turn between α4 and α5 through its hydrogen bonding with the backbone nitrogen of G101 on α5. In hZIP4, N106 likely functions as D98 in pZIP4, because the side chains of both residues have the same length and the oxygen atoms on their side chains can similarly form hydrogen bond with the backbone nitrogen of the conserved glycine residue. On the contrary, a lysine residue (N106K) with a much more flexible and positively charged side chain is very unlikely to play the same structural role as an asparagine or an aspartate does. In addition, unlike C62R severely affecting ZIP4 folding, the corresponding mutation of N106K in mouse ZIP4 does not affect glycosylation and therefore most likely results in a local structural change of the HRD (ref. 32), instead of a global structural perturbation.

In the PCD, the AE-causing mutation P200L (P193 in pZIP4) is in the middle of the H-P linker. The proline residue not only introduces a rigid turn on the H-P linker, but also participates in a hydrophobic core. The flat side chain of P193 is close and parallel to the indole ring of W262* from α12*. The interaction energy of such a stacked proline–tryptophan arrangement has been shown to be quite high (∼7 kcal mol^−1^)^35^. Therefore, the strong interaction between these two highly conserved residues significantly stabilizes the conformation of the H-P linker and α12*. Another AE-causing mutation Q303H (Q299 in pZIP4) is downstream of the PAL motif on α14. The side chain of the universally conserved Q299 is involved in the formation of two hydrogen bonds with the residues on L12–13: its carboxyl oxygen atom with the backbone amide nitrogen of L267 (2.9 Å apart) and its amine nitrogen atom with the backbone carbonyl oxygen of V265 (3.2 Å apart). The lack of Q299 mediated interactions is likely to affect the structure of L12–13. The effects of C309Y (C305 in pZIP4) must go beyond local structural changes because the corresponding mutation in mouse ZIP4 almost completely diminishes glycosylation, suggestive of severe defects in protein folding.

Collectively, mapping of the AE-causing mutations reinforces that both the HRD and the PCD are crucial for ZIP4 function. Particularly, P200L, Q303H and C309Y are clustered at the bridging region, which is one of the most conserved regions in ZIP4-ECD (Fig. 2b), indicating that this structurally flexible region is critical for ZIP4 function, which is consistent with the mutagenesis results (Fig. 5b,c)

### Structure-based sequence alignment of the LIV-1 proteins

**Figure 7:**
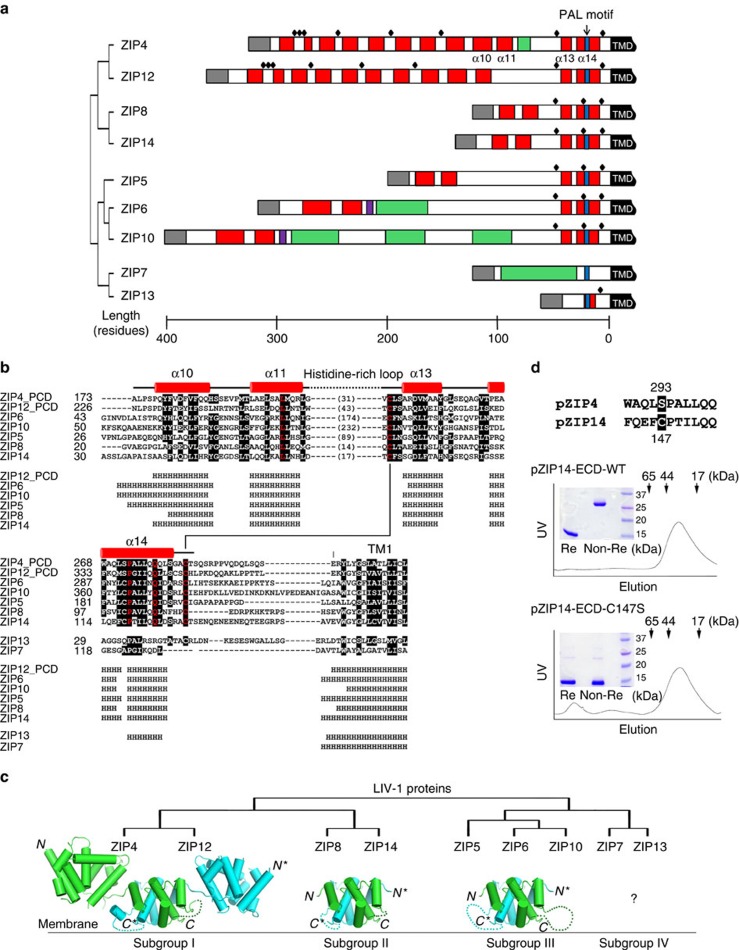
A structurally conserved fold of the ECDs in the LIV-1 subfamily. (**a**) Comparison of the ECDs of the LIV-1 proteins. The phylogenetic tree of the LIV-1 proteins was generated by ClustalW2 (http://www.ebi.ac.uk/Tools/msa/clustalw2/) using the sequences of the full-length human proteins. The sequences of the ECDs from the nine LIV-1 proteins are depicted as rods. The signal peptide is coloured in grey, the predicted helix (by PSIPRED, http://bioinf.cs.ucl.ac.uk/psipred/) in red, the predicted strand in purple, the TMD in black, the histidine rich region in green and the signature PAL motif in blue. Black diamonds indicate universally conserved cysteine residue for each LIV-1 protein. (**b**) Structure-based sequence alignment of the ECDs of the human LIV-1 proteins. The generally conserved residues are shown in black blocks and the invariant residues are highlighted in red (the sequences of ZIP7 and ZIP13 are not included). The secondary structures from pZIP4-ECD are shown above the sequences. The predicted secondary structures of other LIV-1 proteins are shown below the sequence alignment. The National Center for Biotechnology Information (NCBI) reference sequence codes are: NP_570901 for ZIP4, NP_001128667 for ZIP5, NP_036451 for ZIP6, NP_001070984 for ZIP7, NP_001128618 for ZIP8, NP_065075 for ZIP10, NP_001138667 for ZIP12, NP_001121697 for ZIP13 and NP_001128625 for ZIP14. (**c**) The phylogenetic tree of the LIV-1 proteins (top) and the structural models of the ECDs of the LIV-1 proteins (bottom). The predicted unstructured sequences are shown by dotted lines. The length of the dotted line is not proportional to the number of the residues in this region and ZIP4, ZIP6 and ZIP10 contain one or multiple histidine-rich fragments within these regions. (**d**) Characterization of the purified pZIP14-ECD. top: sequence alignment of pZIP4 and pZIP14 in the proximity of the PAL motif. middle: size exclusion chromatography of the wild-type pZIP14-ECD and SDS–PAGE of pZIP14-ECD under reducing (Re) and non-reducing (Non-Re) conditions. bottom: size exclusion chromatography of the C147S mutant and SDS–PAGE of pZIP14-ECD under reducing and non-reducing conditions.

Using the structural information of pZIP4-ECD, we performed structure-based sequence alignment of the ECDs within the LIV-1 subfamily. Surprisingly, the LIV-1 proteins (except for ZIP7 and ZIP13) share a similar dimeric architecture in their ECDs, although the sequences appear to be highly variable (Fig. 7a,b). Sequence alignment reveals that α10, α11, α13 and α14 in ZIP4-ECD are conserved in ZIP5, 6, 8, 10, 12 and 14. Importantly, mapping the conserved residues on the structure of ZIP4-ECD shows that these residues are mostly involved in PCD folding and dimerization (Supplementary Fig. 9), strongly suggesting that these LIV-1 proteins share a similar PCD-like domain which forms a dimer centred at the PAL motif. Based on this analysis, we further classified the LIV-1 proteins into four subgroups (Fig. 7b). In subgroup I, ZIP4 and its close homologue ZIP12 have both the HRD and the PCD. ZIP8 and ZIP14 in subgroup II have the smallest PCD-like domain, as the loop between the first two helices (α10 and α11 in ZIP4) and the last two helices (α13 and α14) is quite short. In contrast, the corresponding region in subgroup III, which consists of ZIP5, ZIP6 and ZIP10, can be longer than 200 residues. Notably, in ZIP6 and ZIP10, this largely unstructured loop harbours clusters of histidine residues, topologically equivalent to the histidine-rich loop in ZIP4-ECD. In subgroup II and III, a common feature is that there is an invariant cysteine residue immediately preceding the PAL motif (Fig. 7a,b). In pZIP4-ECD, this cysteine residue is replaced by a serine residue (S293). As the *C*_β_ of S293 is only 3.7 Å away from the *C*_β_ of S293* on the other protomer (Fig. 3c), the cysteine residue at this position in subgroups II and III is likely to form a disulfide bond covalently linking the two PCD-like domains. To examine this deduction, we expressed and purified the ECD of ZIP14 from *Pteropus Alecto* (pZIP14-ECD) in the same way as pZIP4-ECD. As shown in the size exclusive chromatography profile (Fig. 7d), the elution volume of pZIP14-ECD indicates that the apparent molecular weight is ∼30 kDa, suggesting that pZIP14-ECD most likely forms a dimer in solution (the theoretical molecular weight of pZIP14-ECD is 12.5 kDa). Importantly, in non-reducing SDS–PAGE, pZIP14-ECD immigrates as a 25–30-kDa protein, whereas in reducing SDS–PAGE, it becomes monomeric, indicating the presence of intermolecular disulfide bond(s). To further confirm that the intermolecular disulfide bond forms through C147, which is immediately preceding the PAL motif and corresponding to S293 in pZIP4-ECD, we mutated this cysteine residue to a serine residue. It turns out that the mutant protein is still eluted as a dimer in size exclusive chromatography, but as shown in non-reducing and reducing SDS–PAGE (Fig. 7d), there is no intermolecular disulfide bond any more. Therefore, these data demonstrate that C147 and C147* from each promoter of the pZIP14-ECD dimer must form a disulfide bond at the dimerization interface, as predicted according to the structure-based sequence alignment. Apparently, ZIP7 and ZIP13 (subgroup IV) are outliers as their ECDs cannot be well aligned with the others, except for a degenerated PAL motif.

## Discussion

The distinct physiological functions of the LIV-1 proteins are determined by their specific biochemical properties, tissue distributions, cellular localizations and regulation mechanisms. Besides the highly conserved TMD, the variable ECD is very likely to be a key component determining the unique biochemical properties of each LIV-1 protein. In this work, we present the first crystal structure of a mammalian ZIP4-ECD at a resolution of 2.8 Å. Structural analysis, mutagenesis and cell-based functional studies provide insights into the function of ZIP4-ECD as a critical accessory domain essential for optimal zinc transport, laying out a foundation for further structure–function study of ZIP4 and other metal transporters in the LIV-1 subfamily.

The ZIP family contains thousands of members from bacteria to higher animals, but the large ECD exists only in vertebrates, particularly in mammals. This fact indicates that: (1) the ECD is not absolutely required for zinc transport which is conducted primarily by the highly conserved TMD; (2) the ECD in higher animals must play crucial auxiliary roles. Importantly, the missense mutations on ZIP4-ECD lead to the genetic disorder, AE, strongly suggesting that the ECD is pivotal for ZIP4 function. In this work, we demonstrate that the truncated ZIP4 without the entire ECD loses more than half of the activity when compared with the full-length hZIP4. The significantly reduced zinc uptake is not due to a reduced surface expression level; instead, it is estimated that the *V*_max_ of the truncated hZIP4, after calibration using the quantitated surface expression level, is only ∼20–30% of the *V*_max_ of the full-length hZIP4, indicating ZIP4-ECD is essential for optimal zinc transport (Fig. 1 and Supplementary Fig. 1).

The crystal structure of pZIP4-ECD, which bares 68% homology to human ZIP4-ECD, reveals two structurally independent subdomains, the N-terminal HRD and the C-terminal PCD, connected through a flexible bridging region. The highly conserved PAL motif is interestingly located at the centre of the PCD dimer and the extensive interactions with the neighbouring residues from both protomers strongly suggest that the PAL motif most likely plays a crucial structural role. Consistently, as evidenced by completely diminished glycosylation and dramatically reduced surface expression (Fig. 3e), the P298A and L300A double mutant showed severe defects in folding and trafficking, indicating the critical role of this signature motif in ZIP4 biogenesis. In the HRD, the presence of the four AE-causing mutations (C62R, R95C, A99T and N106K) implies the importance of this subdomain. Indeed, deletion of the HRD (hZIP4-ΔHRD) reduced *V*_max_ by ∼50–60% (Fig. 4), but to a less extent compared with hZIP4-ΔECD (Fig. 1), suggesting that the HRD and the PCD are both contributive to high efficient zinc transport (Fig. 1): the PCD mostly likely plays a structural role by stabilizing ZIP4 dimer, whereas the HRD may play a major and more proactive role in promoting zinc transport (see discussion in the next section). Remarkably, three AE-causing mutations (P200L, Q303H and C309Y) are clustered at the bridging region (Fig. 6) and three structure-guided site-directed mutations (W266A, D275A and Q303A) at this region also result in drastically reduced activity (Fig. 5). Considering that the HRD and the PCD are connected only through the bridging region, the local structural alterations imposed by the mutations in the bridging region may result in global and unfavourable structural changes, accounting for the severe defects in folding and trafficking for W266A, D275A and Q303A. Notably, small but significant amount of D275A and Q303A are expressed on cell surface, but the very low zinc transport activities suggest that the mutant proteins may be trapped in a non-productive conformation, reminiscent of the AE-causing mutation P200L at the bridging region (Fig. 6) which has generally normal surface expression level whereas largely reduced *V*_max_ (ref. 36), reinforcing the key role of the bridging region in zinc transport.

Considering its crucial role in zinc transport, ZIP4-ECD may directly participate in zinc transport as a critical accessory of the zinc transport machinery. According to the putative topology shown in Fig. 3a and the size of a dimeric TMD with a total of 16 transmembrane helices, the HRD and the bridging region are likely to be positioned over the entrance of the zinc transport pathway in the TMD, raising a hypothesis that the HRD and the bridging region may interact with the core component of the zinc transport machinery in the TMD, for instance, via a direct association with the extracellular loops of the TMD, and keep the zinc transport machinery in a functional state (Supplementary Fig. 10a). The pivotal roles of the extracellular loops in the TMD were demonstrated in the study of iron-regulated transporter 1 from *Arabidopsis thaliana*, a prototype of ZIP proteins: the long and highly conserved loop connecting the second and the third transmembrane helices plays vital roles in metal transport activity and substrate selectivity^37^.

Alternatively, ZIP4-ECD may function as an extracellular zinc sensor. By responding to altered metal ion concentration, the soluble domains of some divalent metal transporters proactively regulate metal transport processes^38^,^39^,^40^,^41^,^42^. Interestingly, the ECD of MgtE, a magnesium transporter non-homologous to ZIP4, contains two structurally independent subdomains connected by a flexible linker and dimerizes through the C-terminal subdomain, reminiscent of the overall structure of ZIP4-ECD (Supplementary Fig. 10b). In apo state, MgtE-ECD dimer adopts a similar extended conformation as pZIP4-ECD dimer, and Mg^2+^ binding induces a dramatically structural rearrangement, establishing its function as an Mg^2+^ sensor^41^,^42^. Thus, it would be interesting to examine whether ZIP4-ECD works in a similar way through a rotation of the HRD relative to the dimeric PCD on zinc binding. Considering the flexibility of the bridging region, such a rotation of the HRD is possible. If zinc does switch the functional states of the zinc transport machinery through a coupled movement of the ECD and the TMD, ZIP4 would share a similar autoregulation mechanism proposed for YiiP (ref. 40), a zinc effluxer in cation diffusion facilitator superfamily. Being a zinc sensor would also be consistent with the observation that the AE-causing mutation P200L completely abolishes the extracellular zinc induced internalization of ZIP4, a process in which a zinc sensing mechanism must be involved^36^.

To examine these putative mechanisms and explore other alternative mechanisms, structural studies of the full-length ZIP4 in the absence and presence of zinc ions, as well as functional studies of the purified transporter reconstituted in proteoliposome^43^,^44^, are warranted for future research.

As the PAL motif is highly conserved in the LIV-1 subfamily, there must be common structural features among these proteins^45^,^46^. In this work, we show that pZIP4-ECD forms a homodimer through the PCD subdomain and the signature PAL motif is exactly at the dimerization interface. By introducing a cysteine residue at the identified dimerization interface, we found that the functionally expressed S297C mutant hZIP4, but not the wild-type hZIP4, spontaneously formed homodimer covalently linked via an intermolecular disulfide bond (Fig. 3d). This result confirms the observed PCD-mediated dimerization of pZIP4-ECD, and demonstrates that the full-length hZIP4 forms a homodimer in cells. Interestingly, a recent computational study has suggested that ZIP4-TMD may form a homodimer^47^, and therefore, it is possible that that both ZIP4-ECD and ZIP4-TMD are involved in dimerization. According to the structure-based sequence alignment, all the LIV-1 proteins, except for ZIP7 and ZIP13, share a PCD-like subdomain which forms a homodimer with the PAL motif at the dimerization interface (Fig. 7). Importantly, this hypothesis is strongly supported by disulfide-bonded dimerization of pZIP14-ECD through the cysteine residue preceding the PAL motif (Fig. 7d). Thus, it is very likely that the LIV-1 proteins ZIP5, 6, 8, 10, 12 and 14 also form homodimer through their ECDs in a way similar to ZIP4, and their highly conserved TMDs may also be contributive to dimerization as shown in ZIP13 which has a very short ECD with a degenerated PAL motif^48^.

As central players in maintaining zinc homeostasis, the ZIP proteins play vital roles in many physiological and pathological processes. Establishing the structure–function relationship of the ZIP proteins will greatly improve our understanding about how these zinc influx transporters work and how they are regulated. In this study, the crystal structure of pZIP4-ECD reveals a novel dimeric architecture shared among the LIV-1 proteins. Structure-guided functional study indicates that ZIP4-ECD is essential for optimal zinc transport, which raises a possibility that the activity of ZIP4 can be potentially regulated through ZIP4-ECD by ZIP4 binding proteins, such as tissue plasminogen activator^41^, and zinc or other divalent metals. ZIP4-ECD, particularly the bridging region connecting the two subdomains, can also be an ideal targeting site for ZIP4 inhibitors: as demonstrated in this work, the conformational changes induced by a single mutation at this region may lead to a dramatic slowdown of the zinc transport machinery (Fig. 5).

## Methods

### Genes, plasmids and reagents

The DNAs encoding pZIP4-ECD (Genebank code: ELK11751, residues 36–322) and pZIP14-ECD (UniProtKB/Swiss-Prot: A5D7L5, residues 61–174) were synthesized (Genescript Inc.) with optimized codon for *E. coli* expression, and subcloned into a pLW01 vector (gift from Dr Lucy Waskell at University of Michigan). The DNA sequence of full-length pZIP4 was optimized for human cell expression and also synthesized by Genescript Inc. The complementary DNA of human ZIP4 from Mammalian Gene Collection was purchased from GE Healthcare (GenBank code: BC062625). Both hZIP4 and pZIP4 were subcloned into a modified pEGFP-N1 vector (Clonetech) in which the downstream EGFP gene was deleted and an HA tag was inserted before the stop codon. All the mutations were made using QuikChange mutagenesis kit (Agilent). The sequences of the primers used in the work are listed in Supplementary Table 1. L(+)-Selenomethionine was purchased from Acros Organics. The reagents for protein crystallization were obtained from Hampton Research. Thrombin was purchased from Novagen.

### Cell culture and transfection

Human embryonic kidney cells (HEK293T, ATCC) were cultured in Dulbecco's modified eagle medium (DMEM) (Invitrogen) supplemented with 10% (v/v) foetal bovine serum (Invitrogen) and 1 × Gibco Antibiotic-Antimycotic (Invitrogen). The plasmids were prepared by Qiagen Plasmid Maxi kit (Qiagen) and used to transiently transfect HEK293T cells. Cells were seeded on poly-D-lysine (Sigma) coated 24-well plate and were transfected with Lipofectamine 2000 (Invitrogen).

### Western blot and ZIP4 surface expression assay

The expression of hZIP4-HA (and other constructs and mutants) was detected by western blot using an anti-HA antibody (catalogue# 26183, Thermo Scientific Pierce). hZIP4-HA protein (and other constructs and mutants) expressed at the plasma membrane was indicated by the surface bound anti-HA antibodies recognizing the C-terminal HA tag of ZIP4 proteins^36^,^49^. The cells from multiple subwells of the same 24-well plate for zinc uptake assay were used to determine the surface expression levels of the corresponding proteins. After washing twice with Dulbecco's phosphate-buffered saline (DPBS) on ice, cells were fixed for 5 min in 4% formaldehyde. Cells were then washed three times in DPBS and incubated with 5 μg ml^−1^ anti-HA antibody 1 h at room temperature. Cells were washed five times in DPBS to remove unbound antibodies and then lysed by sonication in SDS–PAGE loading buffer. Cell lysates containing the solubilized anti-HA antibodies bound to the surface ZIP4 were separated using 10% SDS–PAGE, transferred to PVDF membranes. As loading control, β-actin levels were detected using an anti-β-actin antibody at 1:2,500 dilution (catalogue# 4970, Cell Signaling Technology). Bound primary antibody was detected with HRP-conjugated goat anti-mouse immunoglobulin-G (catalogue# 32230, Thermo Scientific Pierce, 1:2,500 dilution) or goat anti-rabbit immunoglobulin-G (catalogue# 7074, Cell Signaling Technology, 1:2,500 dilution) HRP by chemiluminescence. The averaged surface expression levels (from three replicates) quantitated using Image Lab (Bio Rad) programme were expressed as a ratio relative to the surface expression level of the wild-type hZIP4, and then used to calibrate the apparent *V*_max_ obtained in zinc uptake assay (see below). The approach for hZIP4 surface expression detection has been examined for sensitivity and consistency (Supplementary Fig. 11). The uncropped western blots and SDS–PAGE gels are shown in Supplementary Fig. S12.

### ^65^Zinc uptake assays and data analysis

Zinc uptake assays were adapted from ref. 36. as follow, briefly, at 36 h after transfection, cells were washed by zinc uptake buffer containing 10 mM Hepes, 142 mM NaCl, 5 mM KCl, 10 mM glucose, pH 7.3 and then incubated with indicated concentration of ZnCl_2_ (containing 20% ^65^ZnCl_2_) in the same buffer at 37 °C for 20 min with occasional gentle shaking. Uptake was stopped by adding the same volume of stop buffer (ice-cold zinc uptake buffer with 1 mM EDTA). Cells were gently washed two times by zinc stop buffer and lysed in 0.5% Triton X-100. Cell associated radioactivity was measured with a Packard Cobra Auto-Gamma γ-counter and the amount of zinc was calculated using a standard curve generated by plotting ^65^Zn radioactivity against the amount of ^65^ZnCl_2_. The cells transfected with the empty vector were treated in the same way and used as control in the assay. The amount of zinc transported into the cells through ZIP4 was calculated by subtracting the zinc uptake in control cells from the zinc uptake in the cells transfected with ZIP4 gene. The zinc uptake rate is expressed as pmol min^−1^ mg^−1^ protein and the amounts of the protein in the samples were measured using Bradford method (Bio Rad). Curve fitting was conducted using the Hill model (OriginPro 8.5). The apparent *V*_max_ calculated from curve fitting was further calibrated with the quantitated surface expression level and the calibrated *V*_max_s from three independent experiments were combined for statistical analysis. Because of variations of transient expression among different batches of cells, we did not directly combine the values of the calibrated *V*_max_s from three independent experiments. Instead, the calibrated *V*_max_s were normalized by setting the *V*_max_ of the wild-type hZIP4 as 100% and the calibrated *V*_max_s of the other constructs obtained in the same experiment were expressed as a percentage of that of the wild-type hZIP4. Then the normalized and calibrated *V*_max_s from three independent experiments were combined for statistical analysis using Student's *t*-test analysis.

### Protein preparation and crystallization

The expression of pZIP4-ECD was induced by 0.2 mM IPTG at OD_600_∼0.6 in the strain of Origami B(DE3) pLysS (Novagen) in lysogeny broth medium. pZIP4-ECD was purified using nickel-nitrilotriacetic acid (Ni-NTA) column. After removing the N-terminal His_6_-tag by thrombin, the protein was further purified by ion exchange chromatography on a Mono-Q column and then by size exclusion chromatography (GE Healthcare). The purified protein was concentrated to 10 mg ml^−1^ and crystallized by using the sitting-drop method. Leaf-shaped crystals showed up within 2 days in 100 mM MES, 100 mM NH_4_Cl, 17% PGE 3350, pH 6.5 at 21 °C and grew to full size in 1 week. The crystals were cryo-protected by soaking in 30% of PEG 3350 for a few minutes and flash-frozen in liquid nitrogen. The preparation and crystallization of selenomethionine-substituted protein were the same as the native protein. Expression and purification of pZIP14-ECD was the same as pZIP4-ECD.

### Structure determination

The diffraction data of the crystals of native and selenomethionine-substituted protein were collected at 100 K on the beamline of 21-ID-D at LS-CAT in APS, and indexed, integrated and scaled by HKL2000 (ref. 50). The wavelength for data collection was 0.9798 Å. Using single-wavelength anomalous dispersion, the selenium sites and phase were determined by AutoSol in PHENIX (ref. 51). The experimental electron density map is clear enough to trace all the helices. Model building was conducted in COOT (ref. 52) and refinement was performed in Refmac5 in CCP4 suite^53^. Although most of the residues on helices can be unambiguously built into the model, many residues on the loops connecting the helices are still missing after rounds of model building and refinement. The structural model at this stage was then used as search template to solve the structure of the native protein through molecular replacement by using Auto-MR in PHENIX at a resolution of 2.8 Å. After several rounds of model building and refinement, the phase got continuous improvement and more residues, including several highly conserved residues on α2, were built into the final structure model. Ramachandran plot analysis (http://mordred.bioc.cam.ac.uk/~rapper/rampage.php) indicates that, for pZIP4-ECD dimer, 97.6% residues are in favoured region, 1.9% in allowed region and two residues (0.5%) are outliers. All the figures of protein structures were generated by PyMOL v1.3 (Schrodinger LLC).

### Data availability

The atomic coordinates and structure factors for pZIP4-ECD have been deposited in the RCBS, Protein Data Bank under the accession code 4X82. The remaining data that support these findings are available within the article and its Supplementary Information files and from the corresponding author on reasonable request.

## Additional information

**How to cite this article:** Zhang, T. *et al.* Structural insights of ZIP4 extracellular domain critical for optimal zinc transport. *Nat. Commun.* 7:11979 doi: 10.1038/ncomms11979 (2016).

## Supplementary Material

Supplementary Information Supplementary Figures 1-12 and Supplementary Table 1.

## Figures and Tables

**Table 1:** Crystallographic statistics of pZIP4-ECD.

**Data collection**	**Native**	**SeMet**
Space group	P2_1_	P2_1_
Cell dimensions (Å)	*a*=34.2, *b*=68.7, *c*=94.0, *β*=95.8°	*a*=34.0, *b*=68.7, *c*=94.0, *β*=95.8°
*Resolution (Å)	40–2.8 (2.9–2.8)	40–2.85 (2.95–2.85)
*Redundancy	21.7 (18.0)	22.3 (20.1)
*Completeness (%)	99.4 (94.9)	99.8 (98.4)
*<*I/σ*>	20.9 (3.0)	21.9 (3.5)
*R_merge_	0.175 (0.927)	0.132 (0.802)
		
**Refinement**		
Unique reflections	11,017	
Number of atoms	3,078	
*R*_work_*/R*_free_	0.206/0.264	
Averaged *B*-factors (Å^2^)	61.8	
R.m.s. deviations		
Bond lengths (Å)	0.010	
Bond angles (°)	1.42	

^*^Highest resolution shell is shown in parentheses.
