# Supplementary material for: Structural insights of ZIP4 extracellular domain critical for optimal zinc transport
Source: Nat Commun. 2016 Jun 20;7:11979. doi: 10.1038/ncomms11979 (PMC4915132; doi:10.1038/ncomms11979)
Supplement: Supplementary Information — Supplementary Figures 1-12 and Supplementary Table 1. [file ncomms11979-s1.pdf]

Supplementary Information

a

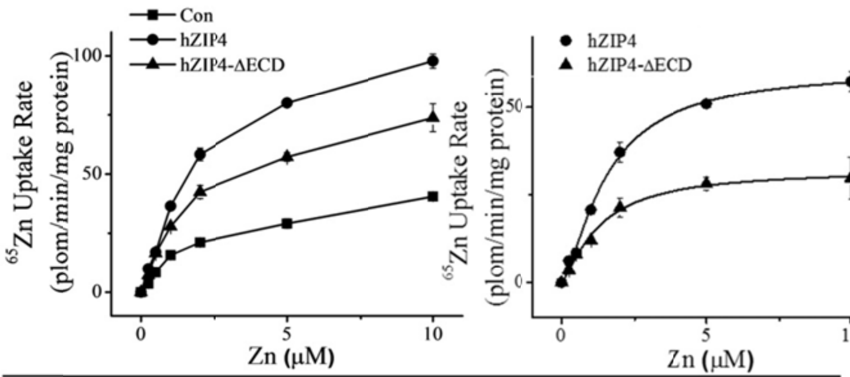

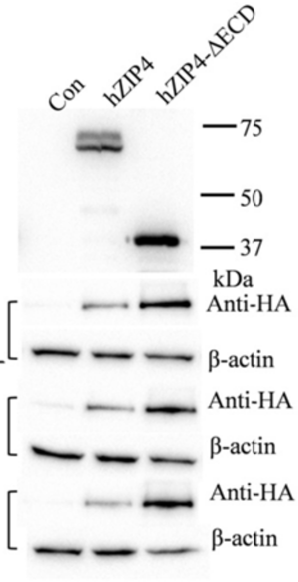

	K_m	n	$V_{max, a}$	Surface	$V_{max, c}$	$V_{max, n}$
hZIP4	1.5 ± 0.2	1.5 ± 0.2	61 ± 3	1	61 ± 3	100%
ΔECD	1.3 ± 0.2	1.3 ± 0.2	32 ± 2	3.5 ± 0.6	9 ± 2	15 ± 4%

b

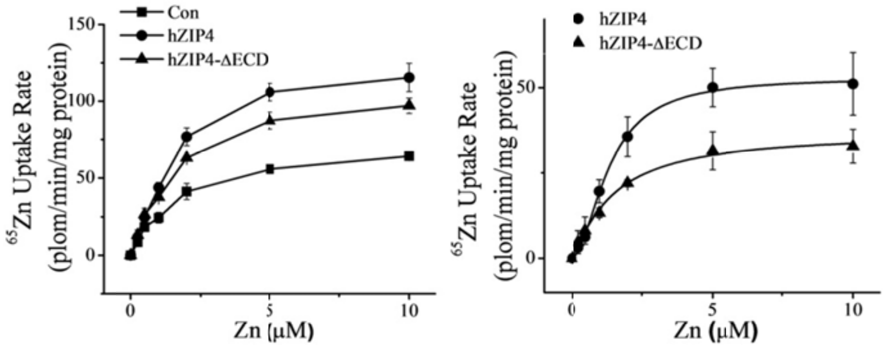

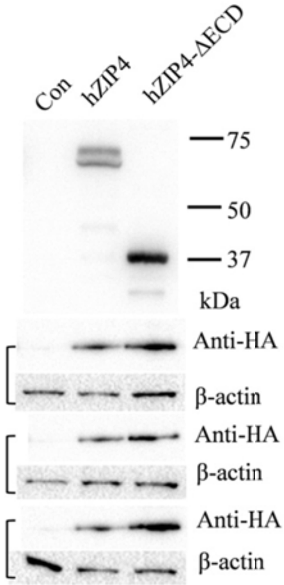

	K_m	n	$V_{max, a}$	Surface	$V_{max, c}$	$V_{max, n}$
hZIP4	1.3 ± 0.1	1.6 ± 0.1	53 ± 1	1	53 ± 1	100%
ΔECD	1.4 ± 0.2	1.2 ± 0.2	37 ± 2	2.2 ± 0.6	17 ± 5	32 ± 10%

Supplementary Figure 1 | Zinc uptake assay of hZIP4 and hZIP4-ΔECD transiently expressed in HEK293T cells. The results of one representative experiment are shown in **Fig. 1** in the main text and (**a**) & (**b**) show the other two independent datasets, respectively. Each dataset includes: (**left**) the raw experimental data; (**middle**) the processed data; (**right**) western blot of the corresponding constructs,

surface expression detection using anti-HA antibody with three replicates, and western blot of β-actin as loading control; (**bottom**) zinc uptake kinetics parameters. The units of K_m and V_{max} are μM and pmol min^{-1} mg^{-1}, respectively. The normalized and calibrated V_{max}s are used for statistical analysis shown in **Fig. 1d**.

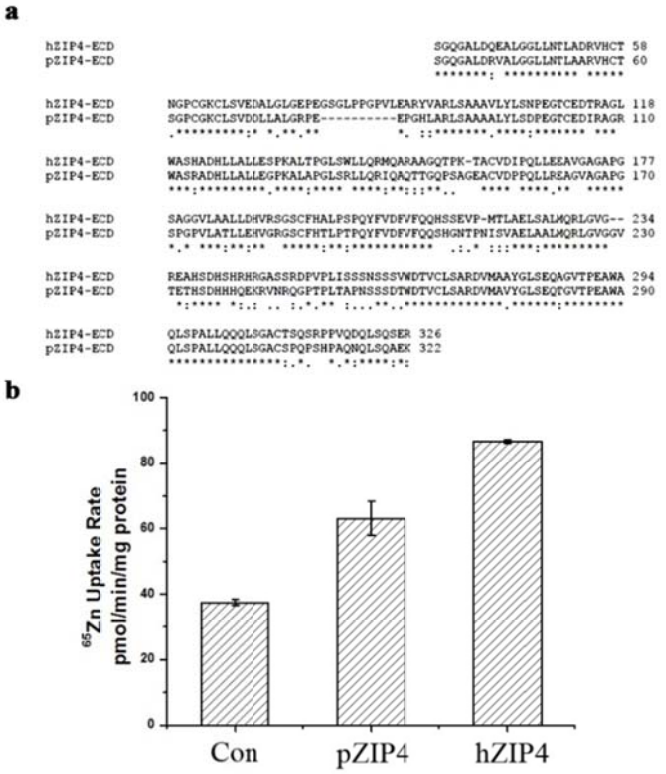

Supplementary Figure 2 | (**a**) Sequence alignment of hZIP4-ECD and pZIP4-ECD. (**b**) Zinc uptake assay of hZIP4 and pZIP4. The full length ZIP4 proteins with C-terminal HA tag were transiently expressed in human kidney HEK293T cells for 36 hours. Cells were incubated with 5 μM $ZnCl_2$ (containing 20% $^{65}ZnCl_2$) in zinc uptake buffer at 37 °C for 20 minutes. The error bars indicated the standard deviations of three replicates in one experiment.

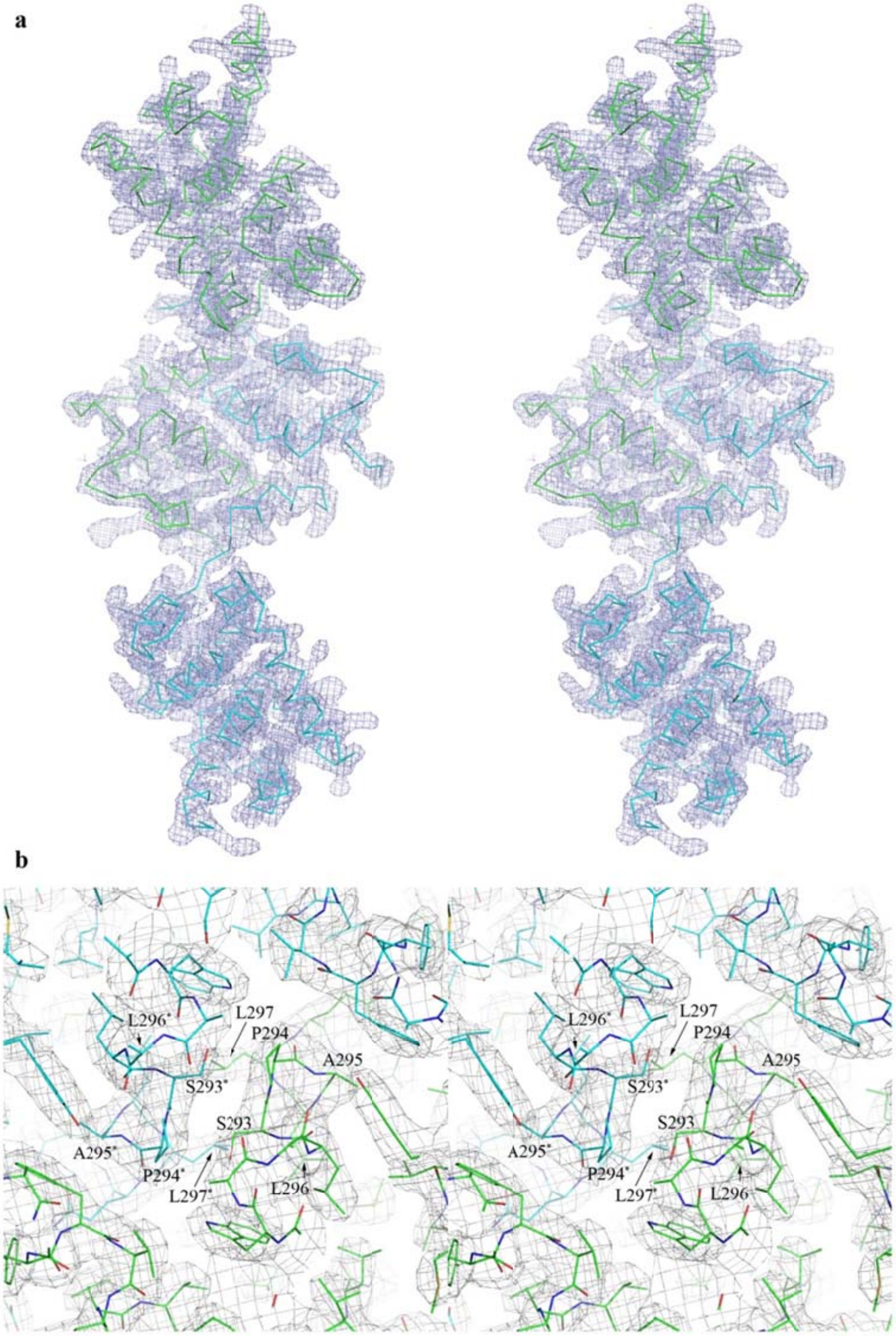

Supplementary Figure 3 | Stereo view of $2F_o$-F_c electron density map (σ=1) in one asymmetric unit of pZIP4-ECD crystal. (**a**) pZIP4-ECD dimer. (**b**) The dimerization interface of the PCD. The residues in the proximity of the "PAL" motif are labeled.

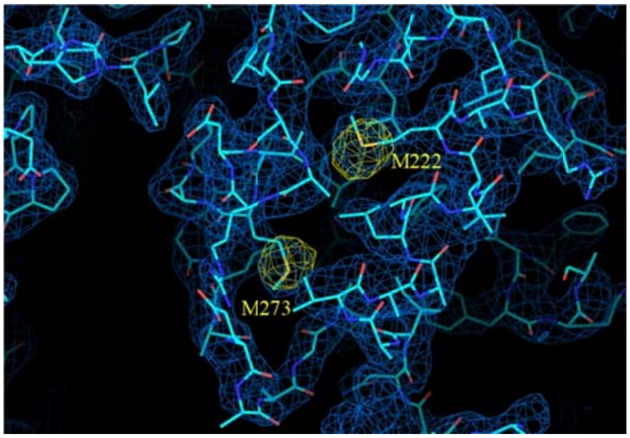

Supplementary Figure 4 | The anomalous difference Fourier map (σ=5, yellow) of selenium atoms in pZIP4-ECD. The $2F_o$-F_c map is shown in blue at σ=1.

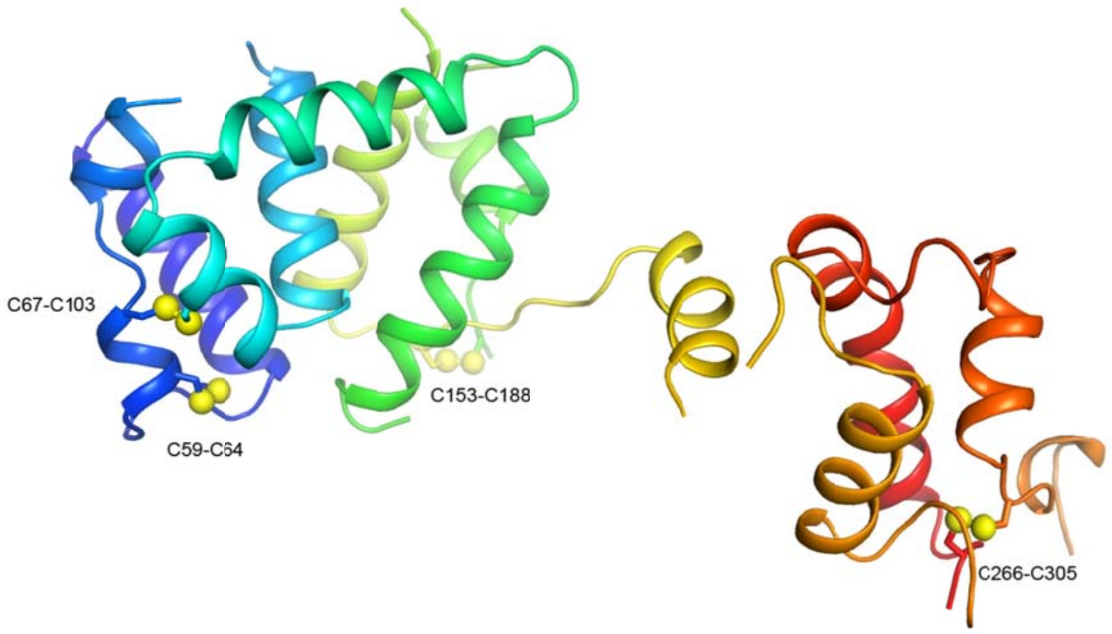

Supplementary Figure 5 | Disulfide bonds in pZIP4-ECD. The monomeric pZIP4-ECD is shown in cartoon mode and the sulfur atoms are depicted as yellow balls.

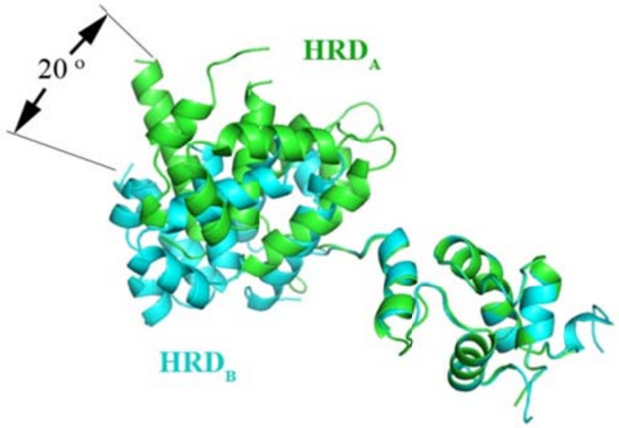

Supplementary Figure 6 | Structural comparison of the two pZIP4-ECD molecules in one asymmetric unit. The PCDs are superimposed to reveal the different orientations of the HRDs relative to the PCDs. The HRD_B in blue rotates 20 degree from the position of the HRD_A in green.

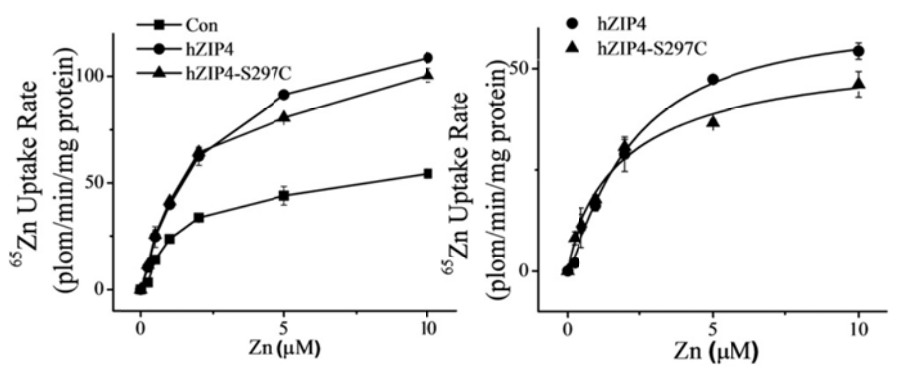

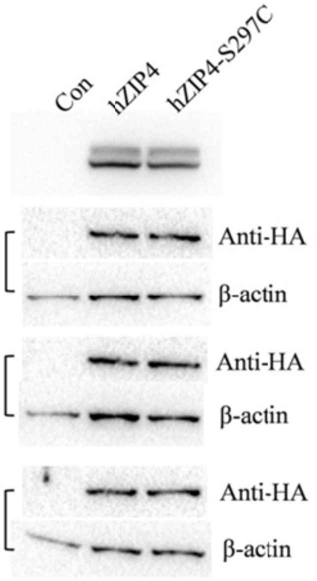

	K_m	n	$V_{max, a}$	Surface	$V_{max, c}$	$V_{max, n}$
hzip4	2.2 ± 0.4	1.3 ± 0.2	62 ± 5	1	62 ± 5	100%
S297C	2.1 ± 1.0	0.9 ± 0.2	56 ± 10	1.0 ± 0.1	56 ± 15	90 ± 25%

Supplementary Figure 7 | Zinc uptake assay of hZIP4-S297C. The results are from one experiment, which include: (**left**) the raw experimental data; (**middle**) the processed data; (**right**) western blot of the corresponding constructs, surface expression detection using anti-HA antibody with three replicates, and western blot of β-actin as loading control; (**bottom**) zinc uptake kinetics parameters (The units of K_m and V_{max} are μM and pmol min^{-1} mg^{-1}, respectively).

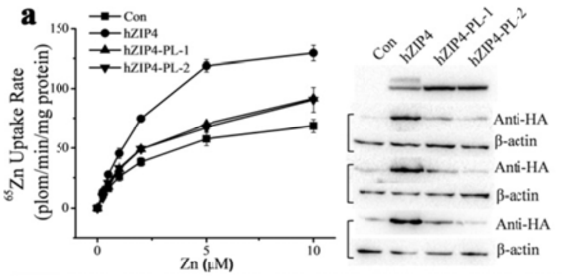

	K_m	n	$V_{max, a}$	Surface
hzip4	2.1 ± 0.5	1.2 ± 0.2	74 ± 11	1
PL-1	-	-	-	0.15 ± 0.08
PL-2	-	-	-	0.06 ± 0.11

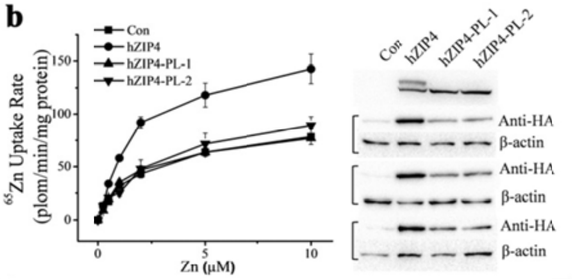

	K_m	n	$V_{max, a}$	Surface
hzip4	1.7 ± 0.7	1.1 ± 0.2	75 ± 15	1
PL-1	-	-	-	0.25 ± 0.06
PL-2	-	-	-	0.25 ± 0.06

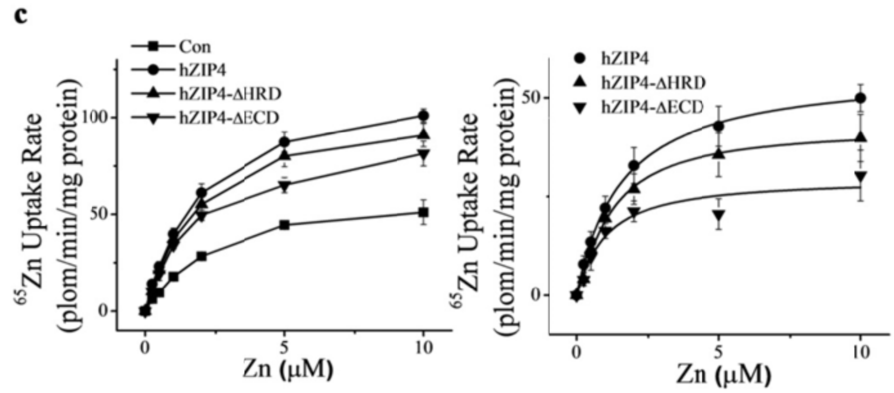

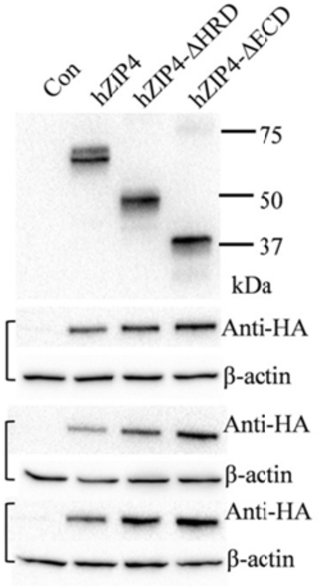

	K_m	n	$V_{max, a}$	Surface	$V_{max, c}$	$V_{max, n}$
hZIP4	1.6 ± 0.1	1.0 ± 0.1	57 ± 2	1	57 ± 2	100%
ΔHRD	1.2 ± 0.1	1.2 ± 0.1	42 ± 2	1.6 ± 0.3	26 ± 6	46 ± 11%
ΔECD	1.0 ± 0.6	1.1 ± 0.5	29 ± 6	2.2 ± 0.6	13 ± 6	23 ± 11%

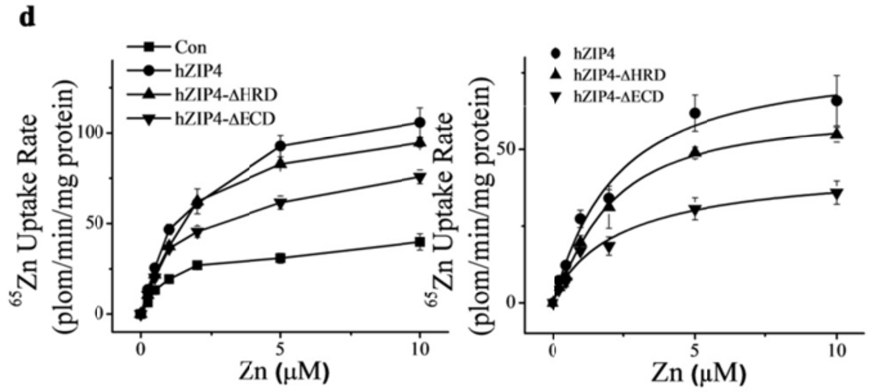

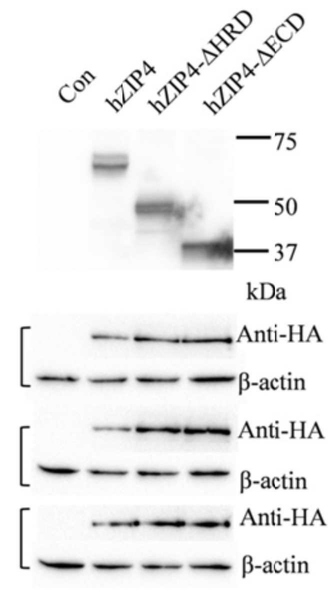

	K_m	n	$V_{max, a}$	Surface	$V_{max, c}$	$V_{max, n}$
hZIP4	2.1 ± 0.8	1.1 ± 0.3	79 ± 13	1	79 ± 13	100%
ΔHRD	1.9 ± 0.2	1.3 ± 0.1	62 ± 2	2.3 ± 0.7	27 ± 9	34 ± 11%
ΔECD	2.4 ± 1.5	1.0 ± 0.3	45 ± 11	2.8 ± 0.8	16 ± 9	20 ± 11%

e

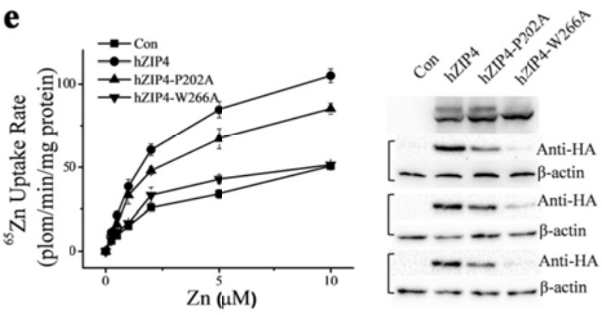

	K_m	n	$V_{max, a}$	Surface	$V_{max, c}$	$V_{max, n}$
hzip4	1.3 ± 0.1	1.4 ± 0.1	57 ± 2	1	57 ± 2	100%
P202A	1.7 ± 0.5	1.3 ± 0.2	39 ± 5	0.62 ± 0.13	63 ± 21	110 ± 37%
W266A	-	-	-	0.07 ± 0.08	-	-

(%)	$V_{max, n}$			Average
	1st	2nd	3rd	
hZIP4	100	100	100	100
P202A	100	110	79	96 ± 16^{NS}

f

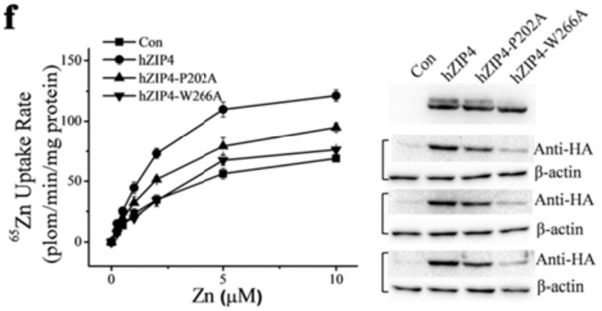

	K_m	n	$V_{max, a}$	Surface	$V_{max, c}$	$V_{max, n}$
hzip4	1.6 ± 0.5	1.2 ± 0.2	62 ± 8	1	62 ± 8	100%
P202A	1.6 ± 0.2	1.2 ± 0.1	29 ± 1	0.59 ± 0.03	49 ± 4	79 ± 7%
W266A	-	-	-	0.09 ± 0.05	-	-

g

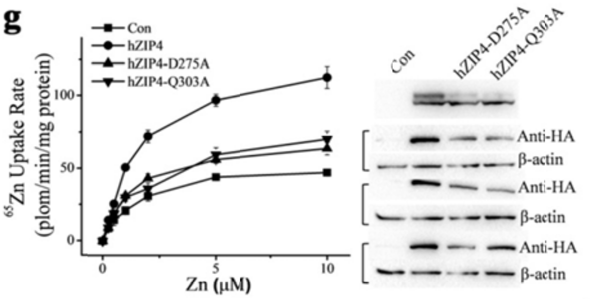

	K_m	n	$V_{max, a}$	Surface
hzip4	1.5 ± 0.3	1.3 ± 0.3	69 ± 7	1
D275A	-	-	-	0.39 ± 0.07
Q303A	-	-	-	0.38 ± 0.17

h

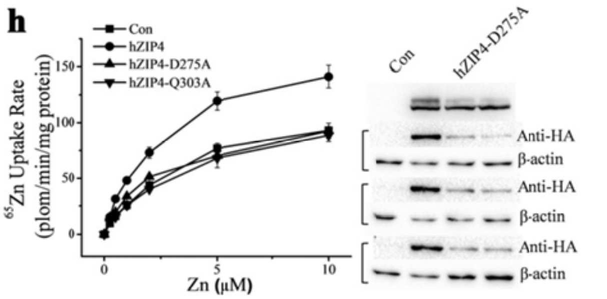

	K_m	n	$V_{max, a}$	Surface
hzip4	1.0 ± 0.2	1.5 ± 0.2	46 ± 6	1
D275A	-	-	-	0.23 ± 0.03
Q303A	-	-	-	0.20 ± 0.06

Supplementary Figure 8 | Zinc uptake assays of ZIP4 constructs and mutants transiently expressed in HEK293T cells. (**a**) & (**b**) WT hZIP4 and the P298A and L300A double mutant (PL). (**c**) & (**d**) WT hZIP4, hZIP4-ΔHRD and hZIP4-ΔECD. (**e**) & (**f**) WT hZIP4, P202A and W266A. (**g**) & (**h**) WT hZIP4, D275A and Q303A. For each construct and mutant, two additional independent datasets are shown here and the results of one representative experiment are shown in **Fig. 3**, **4** and **5** in the main text. Each dataset includes: (**left**) the raw experimental data; (**middle**) the processed data (Note: there is no processed data for some constructs, because their activities are too low to be properly processed); (**right**)

western blot of the corresponding constructs, surface expression detection using anti-HA antibody with three replicates, and western blot of β-actin as loading control; (**bottom**) zinc uptake kinetics parameters. The units of K_m and V_{max} are μM and pmol min^{-1} mg^{-1}, respectively. The normalized V_{max}s are used for statistical analysis shown in the corresponding figures in the main text.

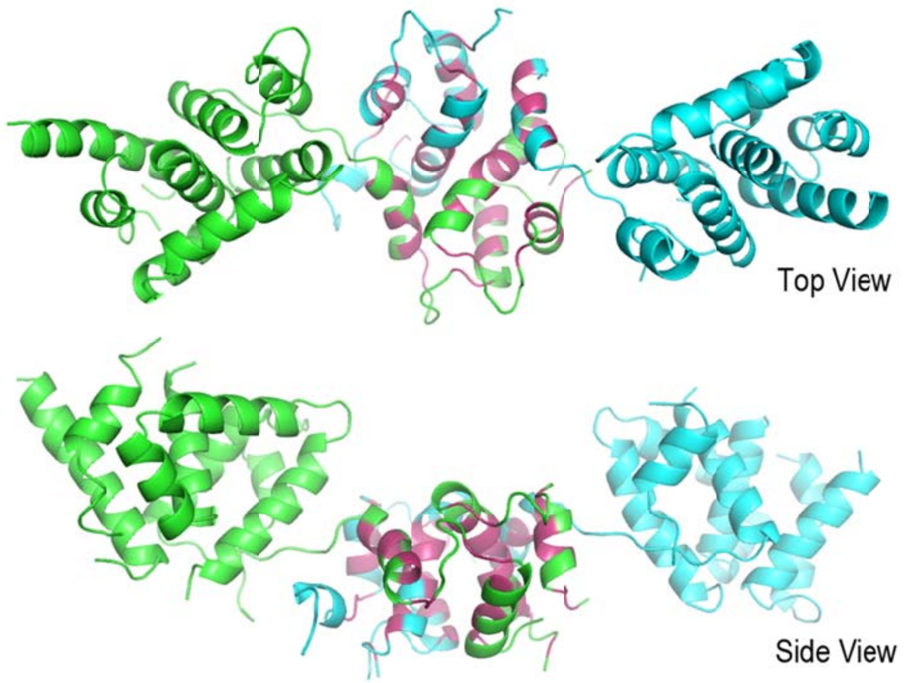

Supplementary Figure 9 | Mapping of the conserved residues (in pink) in the LIV-1 proteins (shown in **Fig. 7b**) on the structure of pZIP4-ECD dimer.

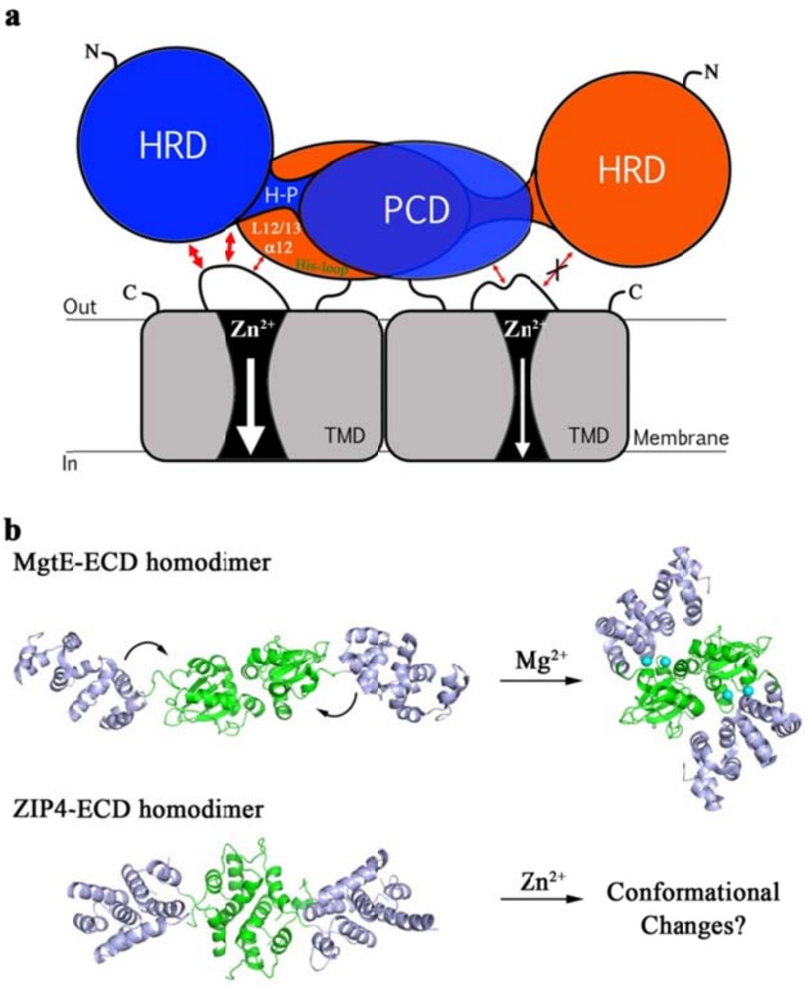

Supplementary Figure 10 | Working hypotheses of ZIP4-ECD. (**a**) ZIP4-ECD functions as an accessory of the zinc transport machinery. In the molecule on the left (blue), the HRD is properly positioned on top of the TMD through the bridging region (H-P linker, α12 and L12 and 13) connecting the HRD and the central dimeric PCD. Through the hypothetic interactions between the HRD and bridging region and the extracellular loops on the TMD (indicated by red arrows), ZIP4-ECD promotes zinc transport by keeping the zinc transport machinery in a functional state. In the molecule on the right (orange), the AE-causing mutations result in altered conformation of the HRD and or the bridging region, disrupting the interactions between the HRD and bridging region and the TMD and leading to less-functional states of the zinc transport machinery. (**b**) ZIP4-ECD functions as an extracellular zinc sensor. Crystal structures of

MgtE-ECD in apo state (left, PDB code 2YVZ) and Mg^{2+}-bound state (right, PDB code 2YVY) are compared with the structure of pZIP4-ECD. Mg^{2+} (cyan balls) binding induced conformational change in MgtE-ECD, indicated by the arrows, establishes its function as an Mg^{2+} sensor. pZIP4-ECD in apo state is also an extended dimeric structure with two structurally independent subdomains linked with a flexible linker. For better comparison, the *N*-terminal subdomain in MgtE-ECD and the HRD in ZIP4-ECD are colored in light blue, and the *C*-terminal dimeric subdomain in MgtE-ECD and the dimeric PCD in ZIP4-ECD are colored in green. The similarity in domain structure and overall structural arrangement raise a hypothesis that ZIP4-ECD may undergo similar conformational changes upon zinc binding at ZIP4-ECD, although ZIP4 shows no homology to MgtE. Through a coupled movement between the ECD and the TMD, zinc ions may regulate the functional status of the zinc transport machinery as proposed in MgtE study (ref 41&42 in the main text).

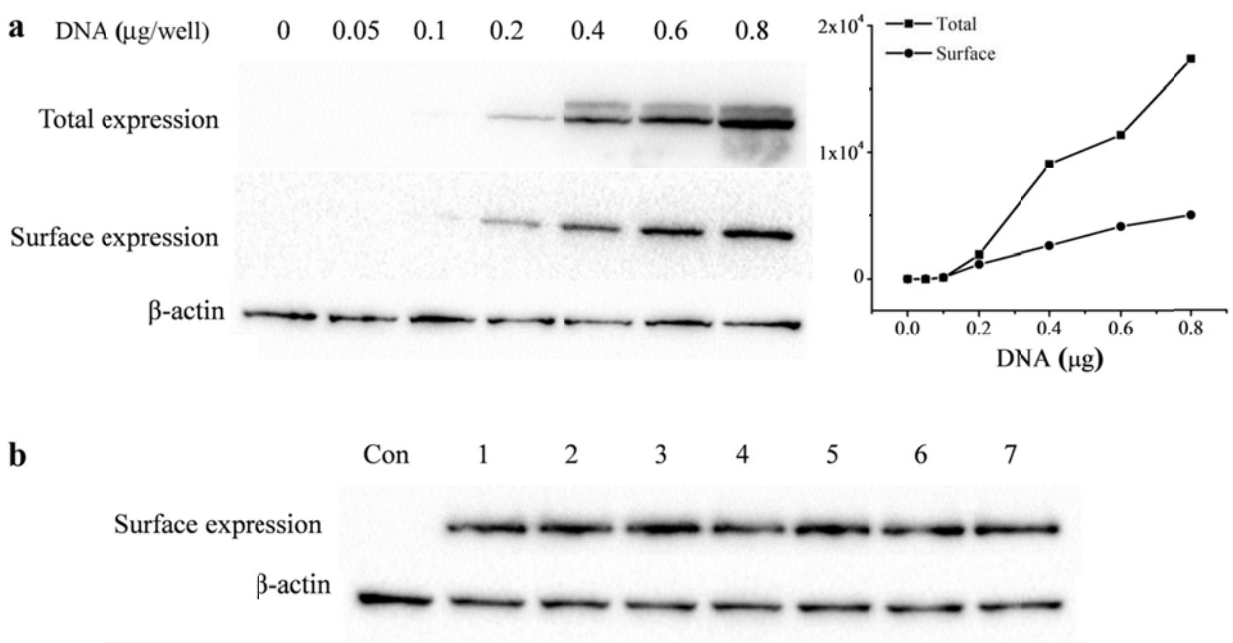

Con	Lane 1	Lane 2	Lane 3	Lane 4	Lane 5	Lane 6	Lane 7	Relative S.D.
0	100 %	110%	121%	95%	120%	105%	106%	10%

Supplementary Figure 11 | ZIP4 surface expression detection. (**a**) Quantitated surface expression levels are correlated with the amount of plasmid DNA used in transfection. Different amounts of plasmid DNA encoding hZIP4-HA were used in transfection with the same DNA to Lipofectamine® 2000 ratio. After 36 hours post transfection, the surface expression levels of hZIP4-HA were detected using anti-HA antibody and quantitated using Image Lab (Bio Rad) program (**left**). As loading control, β-actin in the sample was detected by western blot using anti-β-actin antibody. The generally positive correlation demonstrates the current approach is sensitive enough to quantitatively detect the changes of hZIP4 surface expression level (**right**). (**b**) Consistency of the surface expression level detection. The HEK293T cells cultured in the same 24-well plate were transfected with the same amount of DNA with the same DNA to Lipofectamine® 2000 ratio. After 36 hours post transfection, the surface expression levels of hZIP4-HA were detected using anti-HA antibody and quantitated using Image Lab (Bio Rad) program

(**top**). As loading control, β-actin in the sample was detected by western blot using anti-β-actin antibody. The small relative standard deviation (S.D.) of seven replicates indicates the current approach is reliable to determine quantitated surface expression level for statistical analysis (**bottom**).

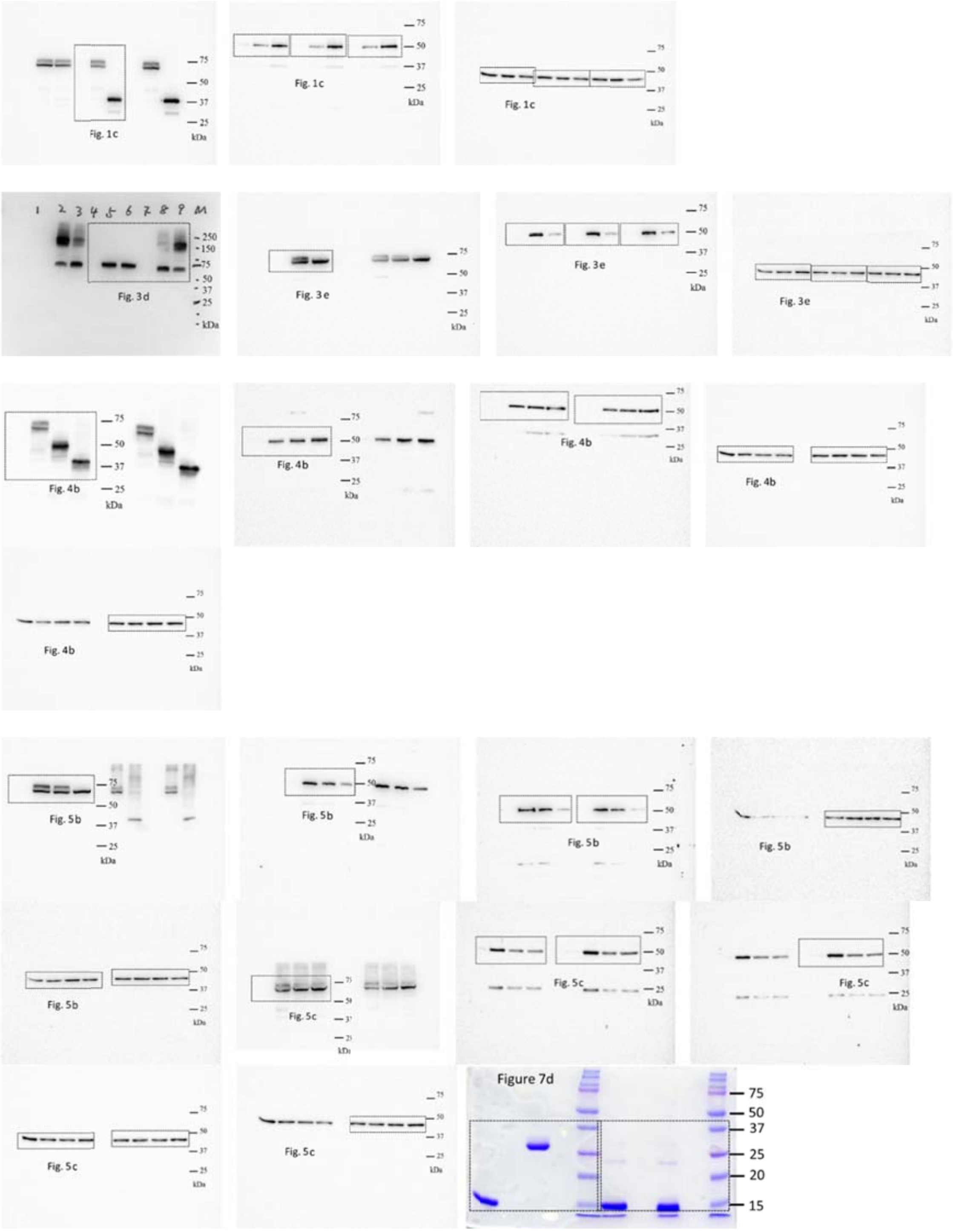
Fig. 1c
Fig. 1c
Fig. 1c
Fig. 3d
Fig. 3e
Fig. 3e
Fig. 3e
Fig. 4b
Fig. 4b
Fig. 4b
Fig. 4b
Fig. 4b
Fig. 5b
Fig. 5b
Fig. 5b
Fig. 5b
Fig. 5b
Fig. 5c
Fig. 5c
Fig. 5c
Fig. 5c
Fig. 5c
Figure 7d
75
50
37
25
20
15
kDa

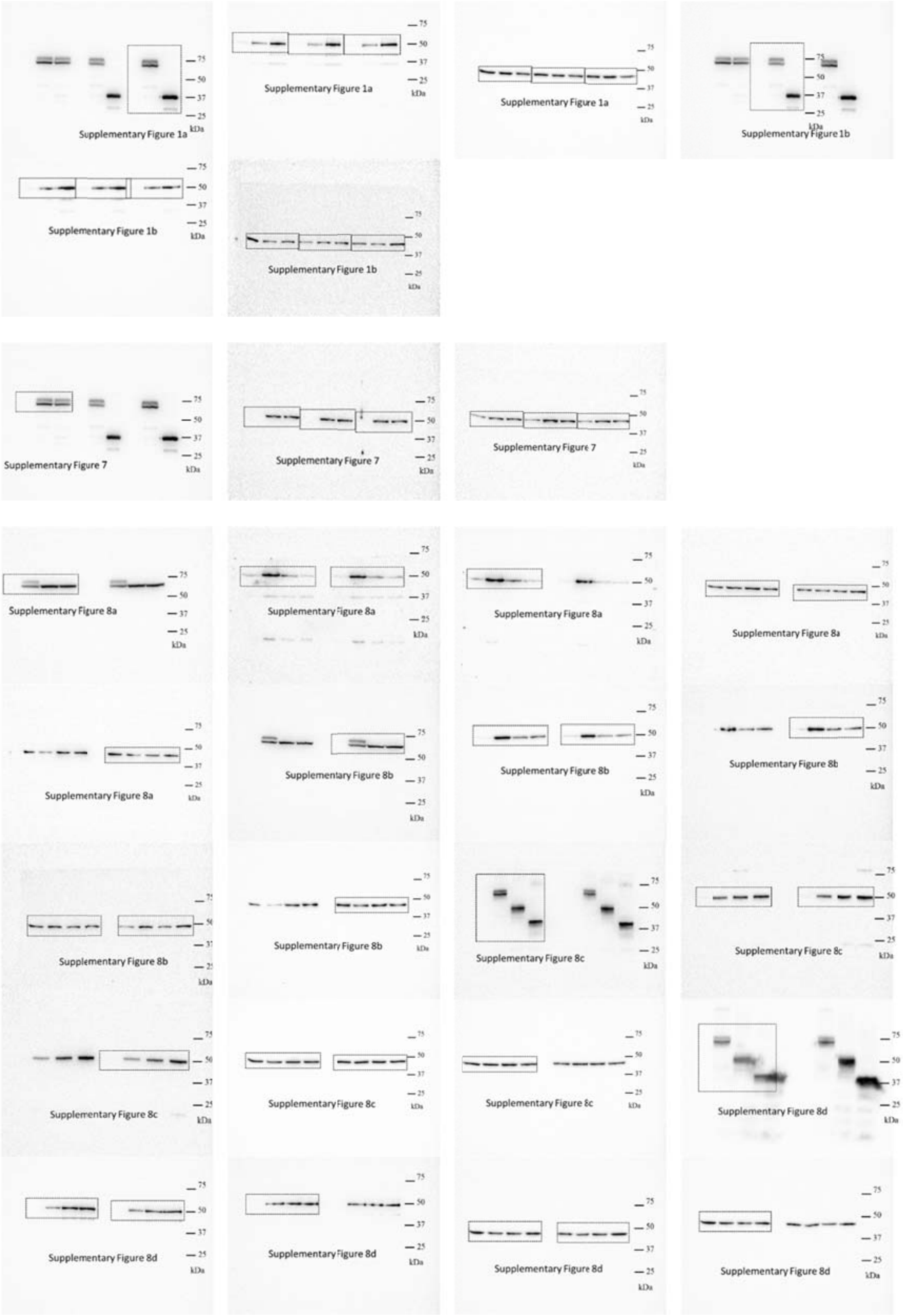
Supplementary Figure 1a
Supplementary Figure 1a
Supplementary Figure 1a
Supplementary Figure 1b
Supplementary Figure 1b
Supplementary Figure 1b
Supplementary Figure 7
Supplementary Figure 7
Supplementary Figure 7
Supplementary Figure 8a
Supplementary Figure 8a
Supplementary Figure 8a
Supplementary Figure 8a
Supplementary Figure 8a
Supplementary Figure 8b
Supplementary Figure 8b
Supplementary Figure 8b
Supplementary Figure 8b
Supplementary Figure 8b
Supplementary Figure 8c
Supplementary Figure 8c
Supplementary Figure 8c
Supplementary Figure 8c
Supplementary Figure 8c
Supplementary Figure 8d
Supplementary Figure 8d
Supplementary Figure 8d
Supplementary Figure 8d
Supplementary Figure 8d
75
50
37
25
kDa

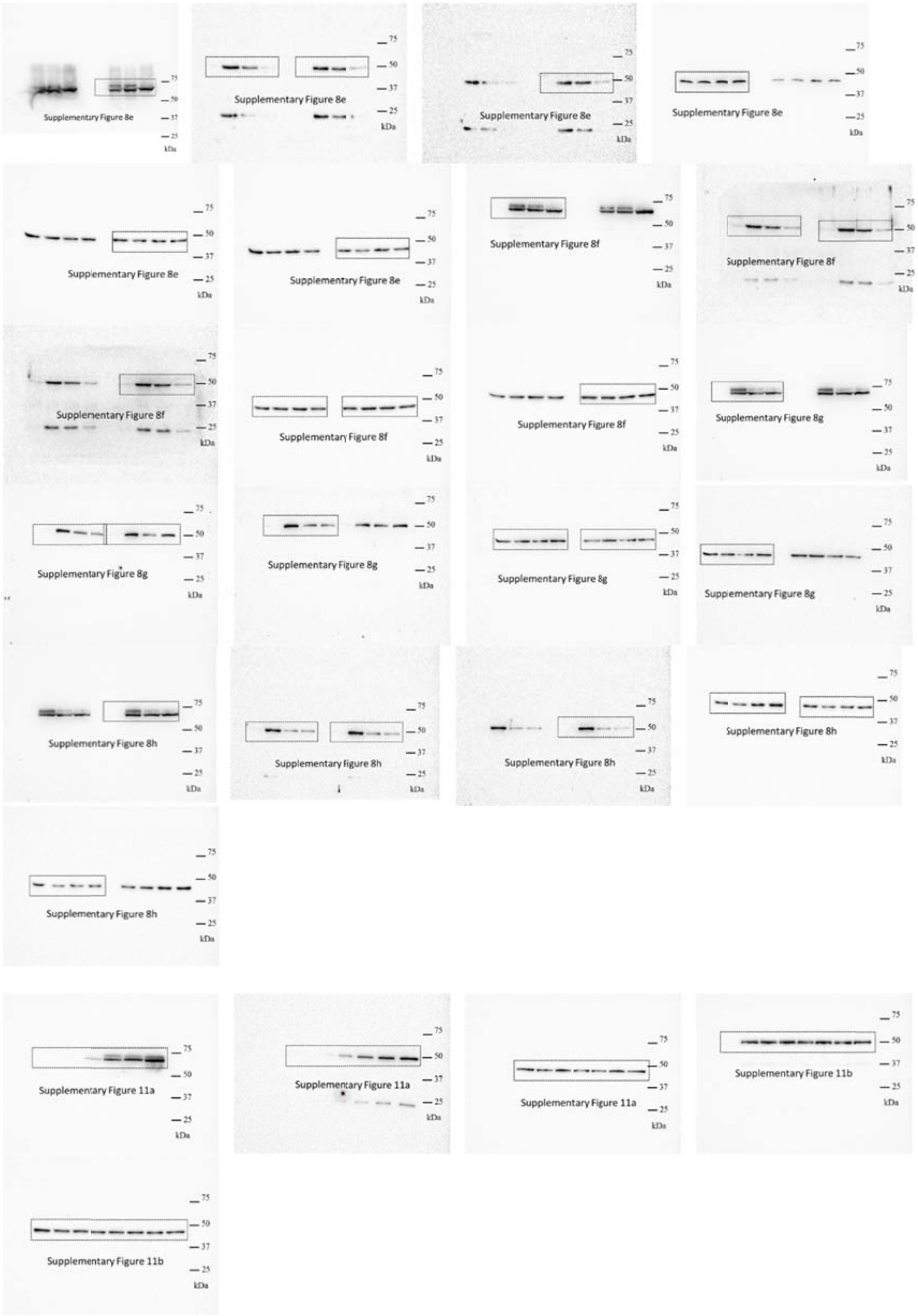

Supplementary Figure 12 | Uncropped Western blots and SDS-PAGE gel in main text and supplementary information.

Supplementary Table 1 | The primers used in this work.

Primer	Sequence
hZIP cDNA ΔHRD fusion reverse	CAAAGTCCACGAAGTACTGAGGGCTCGGCAAGGCGTGGCCCTGGCCAGAGGTGAGCAG 3’
hZIP cDNA ΔHRD fusion forward	CTGCTGGTCTGCTGAGCCTGCTCACCTCTGGCCAGGGCCACGCCTTGCCGAGCCCTCAG 3’
hZIP cDNA ΔECD fusion reverse	GATACCTCTCTGACTGGCTGAGCTGGTCCTGGCCCTGGCCAGAGGTGAGCAGGCTC 3’
hZIP cDNA ΔECD fusion forward	CTGCTGAGCCTGCTCACCTCTGGCCAGGGCCAGGACCAGCTCAGCCAGTCAGAGA 3’
P202A Forward	GCTTCCACGCCTTGCCGAGCGCGCAGTACTTCGTGGACTTTG 3’
P202A Reverse	CAAAGTCCACGAAGTACTGCGCGCTCGGCAAGGCGTGGAAGC 3’
W266A Forward	CAGCAACAGCTCCAGTGTGGCGGACACGGTATGCCTGAGTGC 3’
W266A Reverse	GCACTCAGGCATACCGTGTCCGCCACACTGGAGCTGTTGCTG 3’
D275A Forward	CGGTATGCCTGAGTGCCAGGGCGGTGATGGCTGCATATGGAC 3’
D275A Reverse	GTCCATATGCAGCCATCACCGCCCTGGCACTCAGGCATACCG 3’
P298L300A Forward	GAGGCCTGGGCCCAACTGAGCGCCGCCGCCCTCCAACAGCAGCTGAGTGG 3’
P298L300A Reverse	CCACTCAGCTGCTGTTGGAGGGCGGCGGCGCTCAGTTGGGCCCAGGCCTC 3’
Q303A Forward	CTGAGCCCTGCCCTGCTCCAAGCCCAGCTGAGTGGAGCCTGCAC 3’
Q303A Reverse	GTGCAGGCTCCACTCAGCTGGGCTTGGAGCAGGGCAGGGCTCAG 3’
S297C Forward	CGGAGGCCTGGGCCCAACTGTGCCCTGCCCTGCTCCAACAGCA 3’
S297C Reverse	TGCTGTTGGAGCAGGGCAGGGCACAGTTGGGCCCAGGCCTCCG 3'
